# Gold nanorods enhance different immune cells and allow for efficient targeting of CD4+ Foxp3+ Tregulatory cells

**DOI:** 10.1371/journal.pone.0241882

**Published:** 2021-08-30

**Authors:** Ingrid Safina, Zeid A. Nima Al Sudani, Ahmed Hashoosh, Emilie Darrigues, Fumiya Watanabe, Alexandru S. Biris, Ruud P. M. Dings, Kieng Bao Vang

**Affiliations:** 1 Center for Integrative Nanotechnology Sciences, University of Arkansas at Little Rock, Little Rock, AR, United States of America; 2 Department of Radiation Oncology, Phillips Classic Laser and Nanomedicine Laboratories, Winthrop P. Rockefeller Cancer Institute, University of Arkansas for Medical Sciences, Little Rock, AR, United States of America; Imperial College London, UNITED KINGDOM

## Abstract

Gold nanoparticles (AuNPs) hold great promise in nanomedicine, yet their successful clinical translation has not been realized. Some challenges include effective AuNP targeting and delivery to improve modulation of immune cells of interest while limiting potential adverse effects. In order to overcome these challenges, we must fully understand how AuNPs impact different immune cell subsets, particularly within the dendritic cell and T cell compartments. Herein, we show that polyethylene glycol coated (PEG) gold nanorods (AuNRs) and PEG AuNRs covered with a thin layer of silver (AuNR/Ag) may enhance the immune response towards immune suppression or activation. We also studied the ability to enhance CD4+ Foxp3+ Tregs *in vitro* using AuNRs functionalized with interleukin 2 (IL2), a cytokine that is important in Treg development and homeostasis. Our results indicate that AuNRs enhance different immune cells and that NP composition matters in immune targeting. This knowledge will help us understand how to better design AuNRs to target and enhance the immune system.

## Introduction

Gold nanoparticles (AuNPs) hold great promise for biomedical applications [1–5], including drug delivery and immunotherapy. However, concern over the long-term impact of AuNPs on biological systems has hampered their progress, and their clinical efficacy has not yet been realized [6]. Of particular importance is how AuNPs may interact with the immune system, a vital organ system that protects the body from pathogenic microorganisms and diseases. AuNPs are ideal carriers to target the immune system because they can be easily functionalized with proteins of interest to direct their immune cell trafficking. Immunotherapy-based approaches to target diseased tissues require that intravenously injected AuNPs make contact with immune cells, such as those found in the secondary lymphoid organs. However, AuNP-directed immune targeting can be nonspecific, and uptake is not completely understood. Moreover, the differential impact of AuNPs on distinct immune subsets of the innate or adaptive immune system is unknown. Thus, more in-depth investigations into the effects of AuNPs on immune cell subsets are needed in order for AuNPs’ biomedical potential to be realized.

In response to the presence of pathogens, innate immune cells such as dendritic cells (DCs) can trigger an adaptive immune response by capturing, processing, and presenting antigens. However, DCs are heterogeneous and many subsets exist [7]. For example, the murine DC system contains type 1 interferon-producing plasmacytoid DCs (B220+ pDCs) and conventional DCs (cDCs). The two types of cDCs, lymphoid- and non-lymphoid-derived, have distinct but overlapping functions [7–9]. Lymphoid-derived cDCs can be further classified into 2 subsets: CD8+ and CD11b+ [10].

The adaptive immune response is comprised of B and T cells. For the purpose of this study, we examined CD4 and CD8 T cells, as well as a specialized subset of CD4 helper cells called Tregulatory cells [11–16]. Tregulatory cells express the Interleukin-2 Receptor (IL2Rα or CD25) and factor forkhead box P3 (Foxp3) [15, 17]. Importantly, Tregs (CD4+ CD25+ Foxp3+) unlike non-Tregs (CD4+ Foxp3-), express high levels of IL2Rα, which confers unique sensitivity to low levels of IL2 [18, 19]. Tregs play an important role in preventing autoimmune diseases by suppressing immune responses. Altogether, the cells of the innate and adaptive immune system work in concert to mobilize an immune response, while Tregs limit a response [20]. Moreover, immune cells exist at all portals of NP entry and are most likely the first cells that will come into contact with NPs. However, it is not known how continued exposure to NPs impacts distinct immune subsets. While progress has been made in understanding AuNPs’ impact on the immune system, the work is not exhaustive. Though some studies have used primary immune cells, most have focused on cell lines that do not accurately reflect distinct immune cell lineages *in vivo*. Furthermore, cell lines are relatively homogenous and preclude the assessment of heterogeneous subsets of immune cells that allow for cellular cross-talk, such as those of primary immune cells.

It is acknowledged that nanoparticles themselves, as well as their shape, size [21, 22] surface covering [23], and functionalization [24] with proteins/targeting molecules, can cause different immune cell responses [25, 26]. Recent work has offered clues on how AuNPs affect the immune system [27, 28]. Niikura et al. report that rod-shaped AuNPs coated with West Nile virus envelope proteins (WNVp) were internalized by RAW264.7 macrophages and induced cytokines in an inflammasome-dependent manner [2]. Furthermore, when the authors treated the mice with the WNVp-coated AuNPs, enhanced antibody production was noted in comparison to WNVp alone. However, the interaction of AuNPs with other immune cells was not reported. It is thought that AuNPs can influence the production of cytokines [29] and have innate adjuvant properties. Moreover, others have shown that colloidal gold exposure in mice induced lymphocyte proliferation as well as IL2 production [29].

The pharmacokinetics (PK) and tissue biodistribution of AuNPs ultimately determine their biomedical usefulness. The NP size, surface charge, and surface chemistry influences the PK [30]. Initial studies show that NPs injected into both mice and rats are distributed and found in 25 organs and tissues types but are most notably accumulated in the secondary lymphoid structures, such as the spleen [27, 31]. Since the spleen contains all the major subsets of immune cells, AuNP interactions within this organ need to be studied more in depth. A week after injection with a single dose of AuNPs, the level of Au found in the spleen was ~8.4 ng/g. However, this increased to ~9.1 ng/g within a month and resulted in changes in gene expression associated with the innate immune system [27]. Thus, this single dose caused immune cells to be continuously or chronically exposed to AuNPs for up to a month. Similarly, in a rabbit hepatic tumor model, it was found that 24 hours after injection with AuNPs, the concentration of white blood cells increased [32]. In another study, gold nanoclusters caused acute infection and inflammation in mice [23]. However, the effect of continuous exposure to AuNPs on individual immune cell subsets, e.g., within the DC and T cell lineages, has yet to be fully investigated. Moreover, once the cells of the immune system have taken up NPs, it is not clear how that translates into bioactivity, impairment of immune cell function, and potential cytotoxicity. We published that gold nanorods are taken up by JAWSII DCs, as they were found to be localized in intracellular components, but the extent of these NPs’ impact on distinct immune DC and T cell subsets remains to be investigated [33].

AuNPs are inert, biocompatible, and have been approved for use in a number of clinical studies [34–36]. To take advantage of these NPs’ biomedical potential, we have developed gold nanorods (AuNRs) with various coatings and functionalization, including AuNR covered with a thin (1–2 nm) layer of silver (Ag) and encapsulated with polyethylene glycol [33, 37, 38]. Previously, we demonstrated that this AuNR/Ag nanosystem can impact a bone marrow-derived DC line by upregulating surface marker expression of activation markers [33]. AuNR and AuNR/Ag have similar physicochemical characteristics (size, surface traits, and stability) that have been well characterized, but the impact of immune cell subsets’ continuous exposure to them is not completely understood [33, 38, 39]. To investigate this, we used an *in vitro* system to assess and compare the effect of continuous exposure of both AuNRs and AuNR/Ag on the different cells of the immune system. We found that AuNRs have a different impact than AuNR/Ag on the following murine immune cell lineages: DCs, CD4, CD8 T cells, and CD4+ Foxp3+ Tregs. This finding indicates that the type of AuNR utilized as an immune targeting vehicle matters in nanomedicine.

## Materials and methods

### Gold nanorod synthesis

AuNRs and AuNR/Ag were synthesized, covered with polyethylene glycol (PEG, NANOCS), and fully characterized as published [33, 37–39]. The PEG-coated AuNRs and AuNR/Ag have an aspect ratio (AR) of 3, diameter of 12 nm, and length of 36 nm [33, 37–39]. Please note that herein, the abbreviation “Ag” refers to the chemical element silver. Recombinant murine Interleukin-2 (IL2, R&D Systems) was conjugated to PEG-covered AuNRs to generate a nanosystem that we will refer to as TRIL2. In brief, murine IL2 contains several potential C terminus amines (NH2), which were utilized to attach the IL2 to the AuNRs covered with thiol PEG. We used a standard method to attach the NH2-containing IL2 to the carboxyl-containing PEG substrate via an amide bond using *N*-ethyl-*N*’-(3-(dimethylamino)propyl)carbodiimide and *N*-hydroxysuccinimide.

### Transmission electron microscopy (TEM)

The AuNRs, AuNR/Ag, and TRIL2 were washed with ethanol at least 3 times, then 50 μl were dropped onto a holey carbon-coated copper TEM grid and allowed to dry. A JEOL JEM-2100F TEM with a field emission gun and EDAX EDS system option was used to take images of the AuNRs. The prepared samples were imaged at 80 kV as previously described [33, 37–39].

### Atomic force microscopy (AFM)

AFM was used to image the AuNRs as previously described [33]. For each sample, the solvent (1X PBS) containing 1000 μg/mL of AuNRs was deposited on a silicon (Si) substrate at several spots. The substrate was then dried overnight under a chemical fume hood, leading to well-dispersed AuNRs adhered to the substrate surface. Next, a Bruker FastScan AFM in tapping mode (scan rate: 1 Hz; 256 samples per line) was used to scan the AuNRs. Height and phase images were recorded then polished using Bruker Nanoscope Analysis software (version 1.8).

### Scanning electron microscopy (SEM)

SEM images of AuNR and TRIL2 were obtained by JEOL JSM 7000F with a field emission gun (FESEM). SEM samples were prepared similarly to the AFM samples: a drop of AuNR or TRIL2 suspended in DI water was dripped on a Si wafer chip (approximately 1-cm square) and allowed to dry in a vacuum desiccator.

### UV-visible spectra

UV-Visible NIR absorption spectra were collected with a Shimadzu UV-3600 spectrometer (Shimadzu Scientific Instruments, Inc, Columbia, MD, USA). 1000 μg/ml of either AuNR or TRIL2 was placed in a 1-cm path-length quartz cuvette. The blank or reference was DI water. The spectra were collected from 200–1200 nm at room temperature.

### Zeta potential

Overall surface charge of AuNRs and TRIL2 was measured with a Zeta Potential Reader (Zeta-Reader, ZPi, Mark 21) equipped with darkfield microscopy for enhanced detection. Briefly, the nanoparticles were diluted ~200 μg/ml in DI water and injected inside the chamber, and by electrophoresis, the AuNRs migrated either to the cathode or anode.

### Primary immune cells

C57BL/6 mice (7–10 weeks of age) were sacrificed, the spleens were excised and minced, and single cell suspensions were prepared by passing over a 70-μm filter. Cells were plated at 5.0 x 10^6 cells per well in a 24-well plate and cultured in 10% fetal bovine serum (ATCC, 30–2020), 1% penicillin + streptomycin, 1% L-glutamine, and RPMI-1640 medium (ATCC, 30–2001). Cells were either untreated or treated with 100 μg/ml of AuNR, 100 μg/ml of AuNR/Ag, or 5 μg/ml of lipopolysaccharide (LPS; Sigma) for 3 days. Cells were maintained in a tissue culture incubator at 5% CO_2_, 37°C, and 100% humidity. All experiments and protocols were performed and approved in accordance with the relevant guidelines and regulations set forth by the University of Arkansas at Little Rock Institutional Animal Care and use Committee (IACUC protocol #R-18-05).

### Flow cytometry

After 24 and 72 hours, plated cells were mechanically dissociated from the well plates by washing several times with FACS buffer (1% FBS, 0.1% EDTA, and 1x PBS). Washed cells were stained with Fc block (CD16/CD32, BD Biosciences) followed by cell surface staining with the following antibodies from BioLegend or BD Biosciences: CD11c (N418), CD11b (M1/70), MHC Class II (M5/114/15/2), CD4 (GK1.5), CD8 (53–6.7), CD27 (LG.3A10), CD44 (IM7), CD69 (H1.2F3), and CD197/CCR7 (4B12). Singlets were used in the analysis by doublet exclusion. The data represented throughout the manuscript reflects the live gate based on the Fixable Viability Dye (FVD) APC eFluor 780 exclusion (Thermo Fisher). Fluorescence minus one controls were utilized to determine gating strategy. Cells were acquired using an LSRFortessa (BD Biosciences, Franklin Lakes, NJ) as described [40–42]. The data was analyzed with FlowJo software (TreeStar, Ashland, OR).

### Intracellular staining

Intracellular staining was performed as previously published [41]. Briefly, after surface staining, cell suspensions were stimulated with a cell activation cocktail (BD Biosciences; #423304) and 250 ng/ml of anti-CD3e for 5 hours in a 37°C tissue culture incubator. Next, the suspensions were fixed and permeabilized using a Perm/Wash™ kit per manufacturer’s instructions (BD Biosciences). The following intracellular antibodies were used: IFN-γ (XMG1.2) and Foxp3 (FJK-16S). Intracellular Foxp3 staining was performed as described previously by us [12–14].

### Phosphorylated STAT5 (p-STAT5) staining

Cells were washed, and surface staining was performed. Afterwards, cells were serum-starved for 30 minutes, then either left unstimulated or stimulated with IL2 (R&D systems, 5 μg/ml), AuNR, or AuNR conjugated to interleukin 2 (TRIL2, 5 μg/ml). Cells were then fixed with 1 ml of Fix Perm Buffer III (BD Biosciences), followed by permeabilization with 100% ice-cold methanol for 30 minutes. This was followed by staining for intracellular phosphorylated STAT5 (p-STAT5) for an additional 30 minutes as described previously [12, 13].

### CD25+ Treg isolation

Cell suspensions were made from splenocytes and stained with CD25 PE for 15 minutes at 4°C. Afterwards, stained and washed cells were counter-stained with anti-PE microbeads (CD25 MicroBead Kit, Miltenyi Biotec), and washed cells were loaded onto an LS column on a MACS separator (Miltenyi Biotec). Unlabeled (negative fraction) cells were discarded, while the positive fraction (CD25+ fraction) was eluted using a plunger.

### Cell lysis assay

To detect cell lysis, we used a lactate dehydrogenase kit (LDH kit, Roche). Isolated splenocytes were seeded in 24-well plates at 1.0 x 10^6 cells/well in a final volume of 1 ml and treated with the following concentrations of AuNRs: 1, 50, 100, and 250 μg/ml. Treated cells were incubated overnight at 5% CO_2_, 37°C, 100% humidity. After incubation, 100 μl of the cell suspension was added to a 96-well plate, then 100 μl of the reaction mixture of LDH assay was added, followed by incubation at room temperature for 10–15 minutes. The absorbance was read at 492 nm.

### Cell proliferation assay

Spleens were isolated from C57BL/6 mice, and single cell suspensions were made as described previously [33, 42, 43]. Cells were washed and stained with Cell Proliferation Dye eFlour 670 (CPD eFlour 670, Thermofisher) for 10 minutes at 37°C in the dark. Afterwards, stained cells were washed three times with 1x PBS and resuspended in complete media (10% FBS and RPMI). Cells were plated (1.0 x 10^6 cells/well) and left unstimulated or stimulated with IL2 (5 μg/ml) and with the following conditions for 72 hours: Concanavalin A (5 μg/ml), AuNR (100 μg/ml), and AuNR/Ag (100 μg/ml). After 72 hours in culture, cells were harvested, washed, and stained with FVD eFluor 450 (Thermo Fisher) for 30 minutes, and data was subsequently acquired using an LSRFortessa.

### Statistical analysis

Unless otherwise indicated, all experiments were performed in triplicate with at least 3 technical duplicates per experiment. The data is reported as scatter dot plots with standard error of the mean; *p ≤ 0.05 is significant as determined by a two-sided t test except for in the supplemental figures, where ANOVA was used with a Bartlett’s test and *p ≤ 0.05 is significant.

## Results

### AuNRs increase CD11c+ MHC class II+ dendritic cells

**Fig 1:**
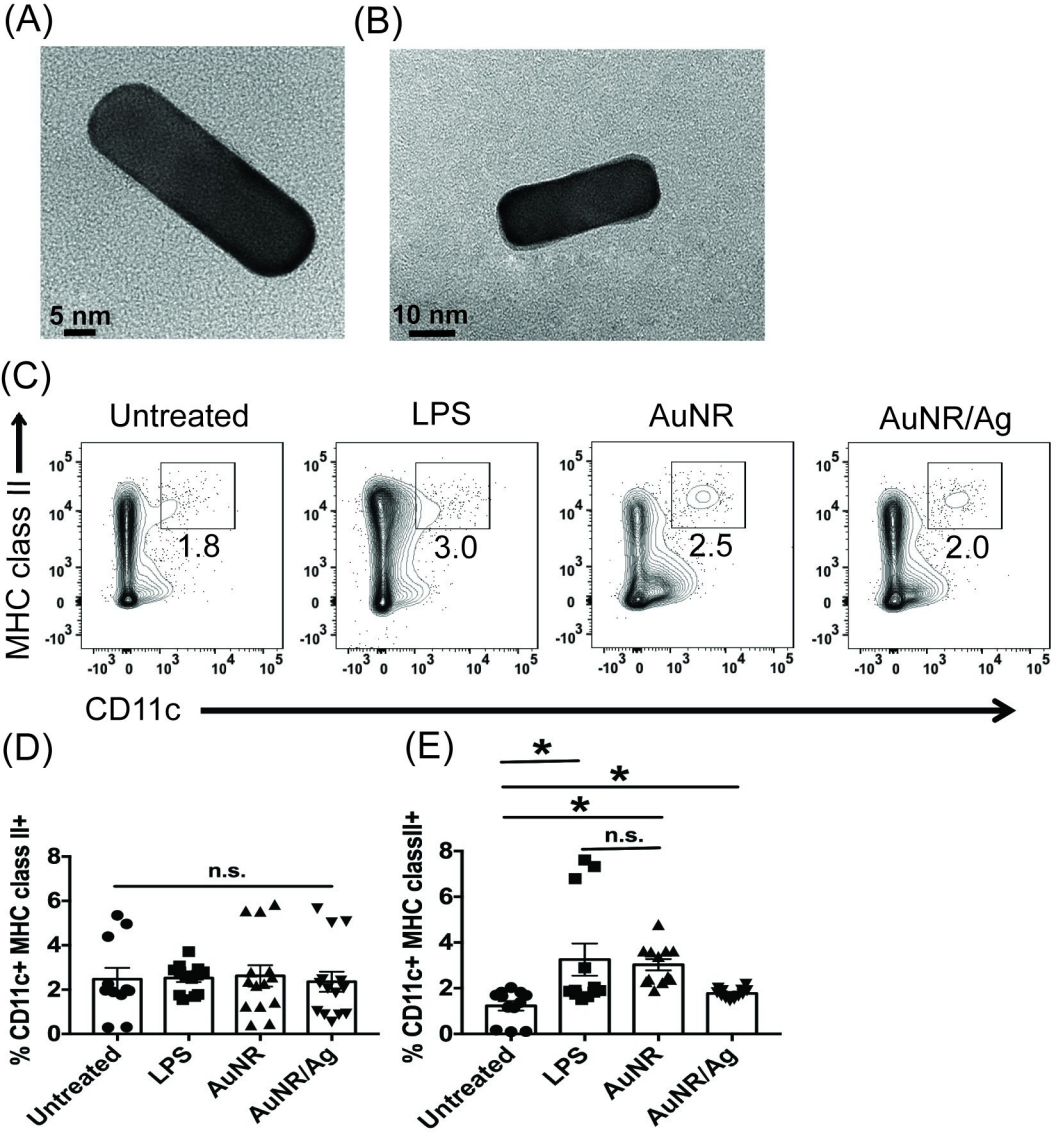
AuNR and AuNR/Ag’s impact on the frequencies of CD11c+ MHC classII+ DCs. TEM images of **(A)** AuNR and **(B)** AuNR/Ag. Whole splenocytes were untreated, treated with LPS (5 μg/ml, positive control), treated with 100 μg/ml AuNR, or treated with 100 μg/ml AuNR/Ag for up to 72 hours. At 24 hours **(D)** or 72 hours **(C)** and **(E)**, cells were harvested and stained for DC markers. The flow data are gated on the percent live cells and are representative of 3 independent experiments with at least 3 technical replicates. The data is shown as scatter dot plots, where error bars represent the standard error of the mean (SEM) and *p ≤ 0.05 is significant as determined by a two-sided t test.

Previously, we demonstrated that 50 μg/ml of AuNRs or AuNR/Ag had little to no effect on cellular viability in an immature (JAWSII) DC line [33]. Furthermore, we found that the AuNRs were endocytosed by JAWSII DCs, as they were found to be localized within membrane-bound, endocytic-like structures [33]. However, because JAWSII is an immature bone marrow-derived DC cell line, these results cannot be generalized to other murine DC subsets. This led us to question which primary DC subsets and T cells may have been impacted and to what extent. In the present study, we used AuNR or AuNR/Ag with an aspect ratio (AR) of approximately 3.0 ± 0.23 (Fig 1A and 1B); these AuNRs have been fully characterized as published [33, 37–39].

To assess the immunotoxicity of AuNRs and AuNR/Ag, we used heterogeneous primary immune cells derived from splenocytes containing the DC subsets of interest and CD4 and CD8 T cells. Though other lymphoid organs such as the lymph nodes are known to contain these immune subsets, the spleen is easily accessible and efficiently provides us with the large number of cells required for experimentation. We made cell suspensions from the spleen of mice and treated them with AuNRs or AuNR/Ag at the following concentrations: 1, 50, 100, and 250 μg/ml. After 24 hours, we performed an LDH assay to assess cell lysis. We found that up to 100 μg/ml of AuNR and AuNR/Ag had a minimal impact on the cell lysis of splenocytes (S1 Fig). At concentrations over 250 μg/ml, the AuNR/Ag was toxic to primary immune cells (S1 Fig). Thus, for the rest of the *in vitro* experiments, we utilized ~100 μg/ml of either AuNR or AuNR/Ag.

To examine the impact of AuNRs on lymphoid DCs, the DCs needed to be easily accessible. The spleen contains the bulk of lymphoid-derived DC subsets and plasmacytoid DCs (pDCs) in question. In this study, we focused on the following murine DCs: pDCs and lymphoid-derived DCs (CD8+ and CD11b+). Furthermore, we were interested to see how continuous exposure to AuNRs impacted DCs over time without the influence of exogenous cytokines. To study this, we treated splenocytes with 100 μg/ml of AuNR, 100 μg/ml of AuNR/Ag, or 5 μg/ml LPS (positive control), and cells were cultured for up to 72 hours. At 24 and 72 hours, cells were harvested and stained for DC subsets. Interestingly, after 24 hours, neither AuNR nor AuNR/Ag had induced any difference in the percentage of CD11c+ MHC class II+ DCs (Fig 1D). However, at 72 hours, the percentages of AuNR- and AuNR/Ag-treated CD11c+ MHC class II+ DCs had increased compared to the untreated samples, and this increase was statistically significant (Fig 1C and 1E). By 72 hours, the LPS positive control results were significant compared to untreated cells but not significant compared to AuNR-treated CD11c+ MHC class II+ DCs (Fig 1E).

Next, we examined if the increased percentages of CD11c+ MHC class II+ DCs corresponded with an increase in the cDC subsets (CD8+, CD11b+) or pDCs (B220+ DCs). After 24 and 72 hours, we found no difference in any of the cDC subsets or pDCs (S2 Fig). In summary, we found that while increased percentages of AuNR- and AuNR/Ag-treated splenocytes enhanced the percent of CD11c+ MHC class II+ DCs, this did not correlate with increases in the other DC subsets.

### AuNR/Ag impact on CD8 T cells

**Fig 2:**
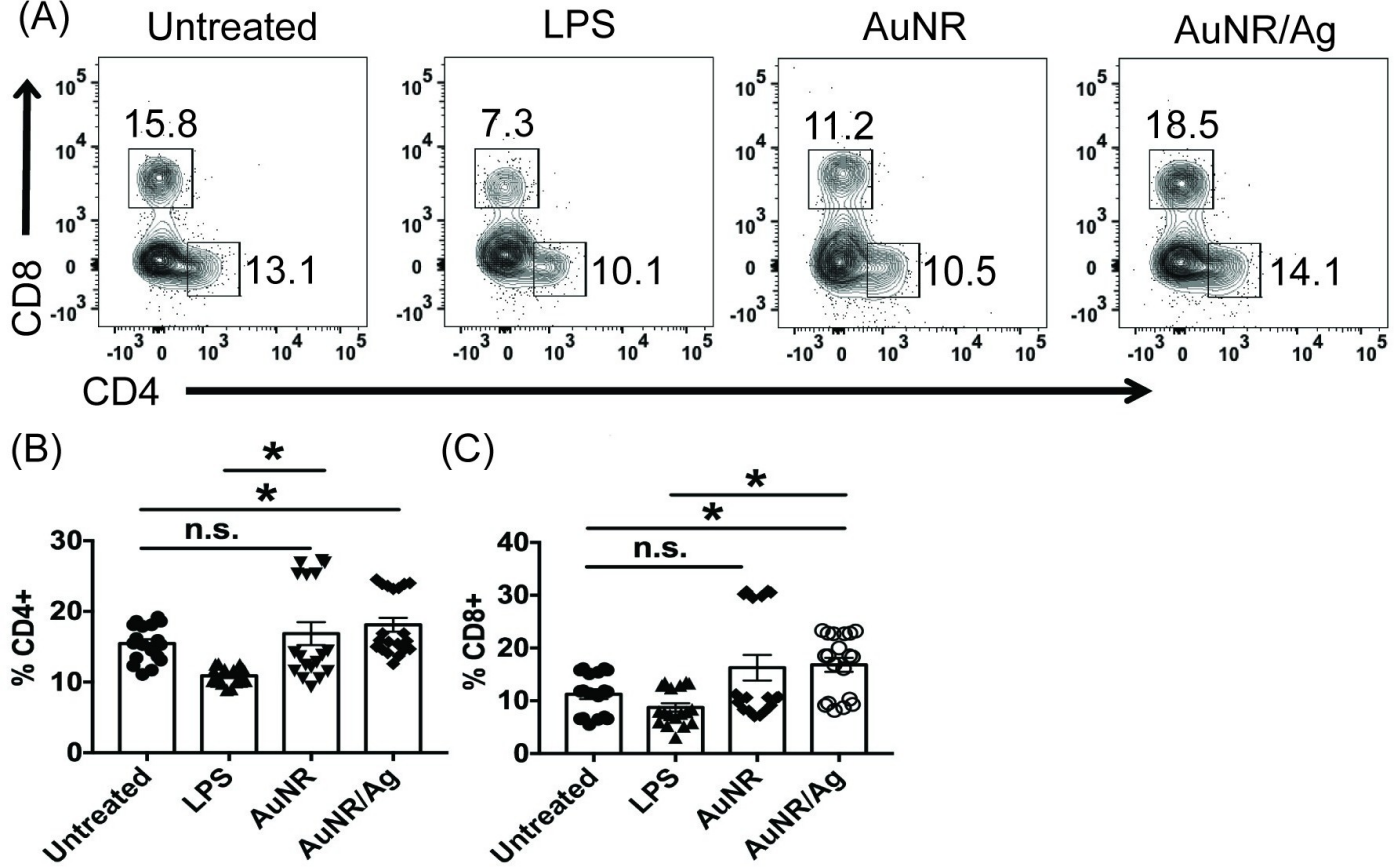
AuNR and AuNR/Ag have different impacts on CD4+ and CD8+ T cells. Splenocytes were treated with 5 μg/ml of LPS or 100 μg of either AuNR or AuNR/Ag for 72 hours, and flow cytometry was performed. The flow data are gated on the percent live, and the combined data represent 3 independent experiments with at least 3 technical replicates. The data is represented as scatter dot plots, where error bars represent the standard error of the mean; *p ≤ 0.05 is significant as determined by a two-sided t test.

Since the AuNRs increased the percentages of CD11c+ MHC class II+ DCs, we wanted to assess their impact on CD4 and CD8 T cells. To do so, we treated splenocytes with either LPS (5 μg/ml) or 100 μg/ml of AuNR or AuNR/Ag for up to 72 hours. After 24 hours, we found that both AuNR and AuNR/Ag seemed to have a slight “edge” in increasing percentages of CD8+ T cells (S3 Fig). However, by 72 hours, this edge only appeared in the AuNR/Ag, and it was significant compared to untreated CD8+ T cells (Fig 2C). When we examined the effect on CD4 T cells, we found that by 24 hours, there was no difference in terms of AuNR-, AuNR/Ag-, or LPS-treated CD4+ T cells compared to untreated cells (S3 Fig). However, by 72 hours, AuNR/Ag-treated splenocytes had the higher percentage of CD4+ T cells, which was statistically significant compared to untreated controls (Fig 2B).

We next examined whether this increased T cell percentage was due to an increase in T cell activation. We treated the cells as described earlier then stimulated them and stained for CD44, CCR7, and CD69. When we examined the expression of CD44 and CCR7 on CD8 T cells, we found no differences in the percentages of CD8+ CD44+ or CD8+ CCR7+ T cells compared to their untreated counterparts (S4 Fig). However, when we examined CD69 expression on CD8+ T cells, both AuNR and AuNR/Ag had increased percentages of CD8+ CD69+ T cells by 72 hours compared to 24 hours (S5A and S5B Fig). Next, we investigated whether AuNR impacts CD8+ T cell function. As a marker of function, we stained CD8+ T cells to assess their production of IFNγ. After 24 hours, AuNR- or AuNR/Ag-treated CD8+ T cells were IFNγ+ but were similar to untreated controls (S5D Fig). However, by 72 hours, AuNR-treated cells had a statistical reduction in CD8+ IFNγ+ T cells compared to untreated cells (S5E Fig).

### AuNRs enhance CD4+ Foxp3+ Tregs

**Fig 3:**
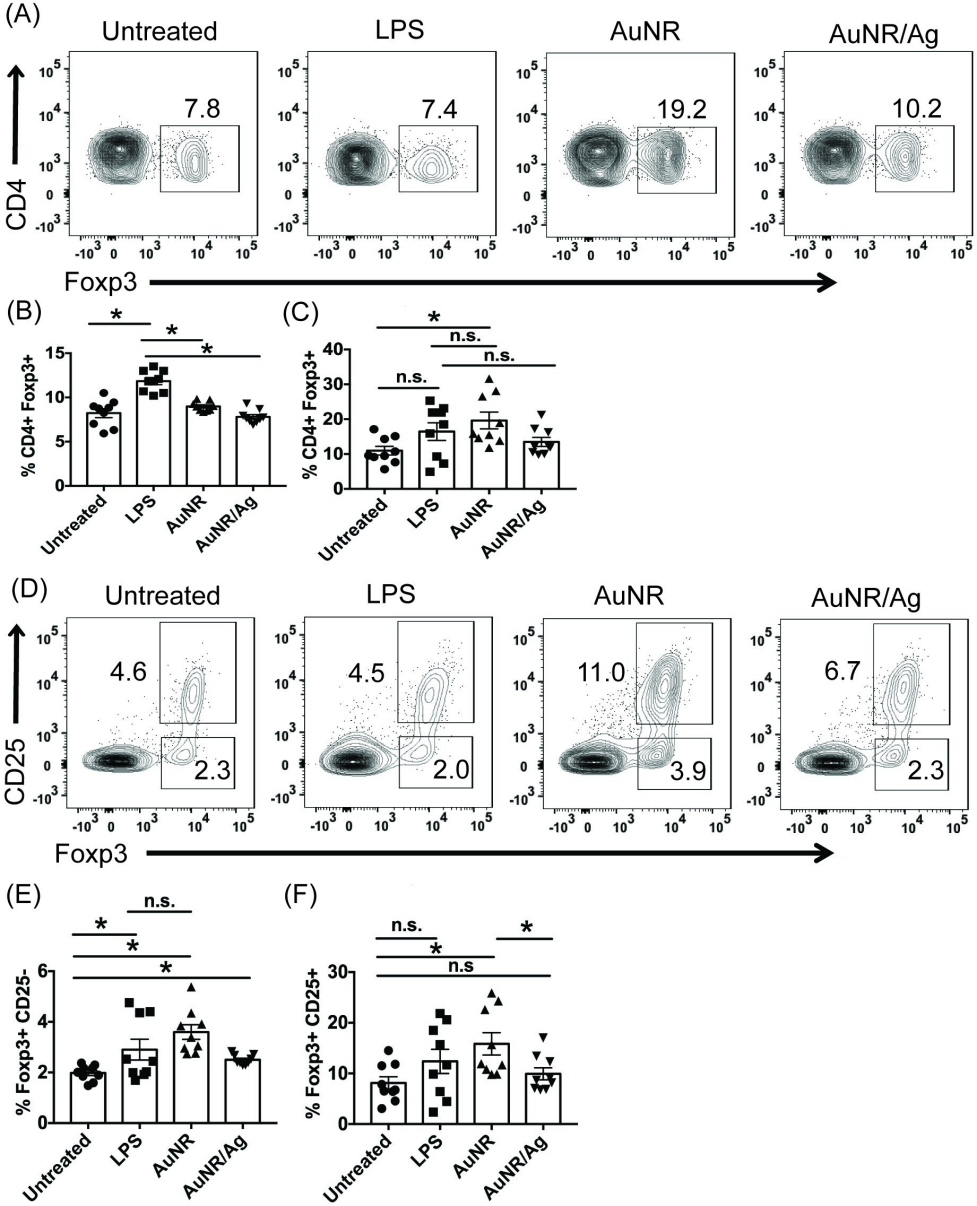
AuNR and AuNR/Ag have different impacts on CD4+ Foxp3+ Tregs. Splenocytes were either untreated or treated with 100 μg/ml AuNR, 100 μg/ml AuNR/Ag, or 5 μg/ml LPS, and flow cytometry was performed after 24 hours **(B)** and **(E)** or after 72 hours: **(A), (C), (D),** and **(F)**. The flow data are gated on the percent live, and the experiments are a compilation of 3 independent experiments; n = 9 per group. The data is represented as scatter dot plots, where error bars represent the standard error of the mean and *p ≤ 0.05 is significant as determined by a two-sided t test.

Next, we investigated if AuNRs can impact CD4+ Foxp3+ Tregs. When splenocytes were treated with AuNR or AuNR/Ag, the former enhanced the frequency of CD4+ Foxp3+ Tregs, while the latter did not. This trend emerged after 24 hours and continued up to 72 hours, and this was significant compared to the untreated CD4+ Foxp3+ Tregs (Fig 3A–3C). It has been recognized that IL2-dependent signals are required for CD4+ Foxp3+ thymic Treg generation [12–14, 15]. IL2 signals via a tripartite receptor composed of the IL2Rα (CD25), beta (β), and γc subunit, making this pathway an important therapeutic target for driving the development of CD4+ Foxp3+ Tregs. Examining the expression of CD25 on Foxp3+ cells, we found that a majority of CD4+ Foxp3+ Tregs were CD25+, with a smaller subset being CD25- (Fig 3D–3F). However, both the CD4+ CD25- Foxp3+ and the CD4+ CD25+ Foxp3+ Treg populations were significant in the AuNR-treated groups compared to untreated splenocytes that contained the same subsets (Fig 3E and 3F). Notably, there was no difference between the LPS-treated controls and the AuNR-treated CD4+ CD25- Foxp3+ and CD4+ CD25+ Foxp3+ Tregs (Fig 3E and 3F). Overall, AuNR enhanced frequencies of both CD4+ CD25- Foxp3+ and CD4+ CD25+ Foxp3+ Tregs (Fig 3D–3F).

**Fig 4:**
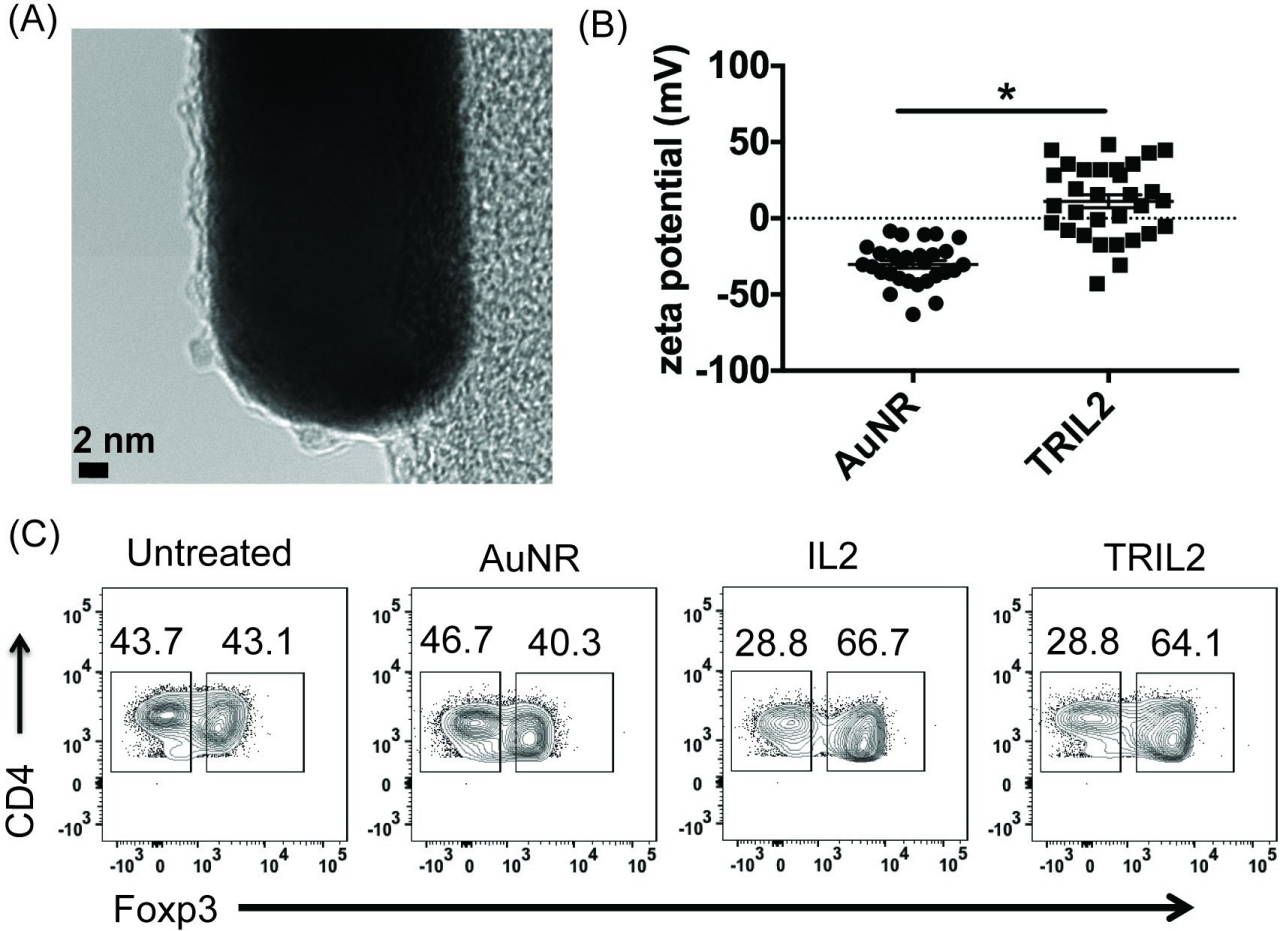
AuNRs functionalized with IL2 enhance CD4+ Foxp3+ Tregs. **(A)** TEM image of AuNRs conjugated to IL2 (TRIL2). **(B)** Zeta potential of AuNR and TRIL2. **(C)** Magnetically isolated CD25+ splenocytes treated with 5 μg/ml of either AuNR, IL2, or TRIL2. The flow data are gated on the percent live, and the data shown is a compilation of 3 independent experiments; n = 9 per group. The data is represented as scatter dot plots, where error bars represent the standard error of the mean and *p ≤ 0.05 is significant as determined by a two-sided t test.

Development of thymic CD4+ Foxp3+ Tregs requires signaling through the IL2-IL2 receptor-STAT5 pathway [14, 19, 43]. Thus, IL2-induced STAT5 signaling is important in natural (n) CD4+ Foxp3+ Treg development [13, 14, 44]. In contrast, among others, peripheral (p) Tregs require both IL2 and transforming growth factor-β signals [45]. Of note, we do not make a distinction between nTregs and pTregs in this manuscript. To partially examine the effect of AuNR’s influence on CD4+ Foxp3+ Tregs, we conjugated IL2 to AuNR, thus creating TRIL2 (Fig 4A and 4B). Successful conjugation of IL2 to the AuNRs was confirmed by zeta potential (Fig 4B). UV-Vis further confirmed that IL2 was conjugated to the AuNRs by an absorbance peak at 280 nm (S6 Fig). We also imaged TRIL2 using AFM and SEM (S7 Fig). Next, we investigated if TRIL2 could influence splenic CD4+ Foxp3+ Tregs. Because CD25 marks the majority of CD4+ Foxp3+ Tregs, we isolated CD25+ Tregs with CD25 microbeads and treated them overnight with TRIL2 or AuNR alone and stained for CD4+ Foxp3+ Tregs. We found that, in contrast to AuNR alone, TRIL2 was able to increase frequencies of CD4+ Foxp3+ Tregs (Fig 4C).

**Fig 5:**
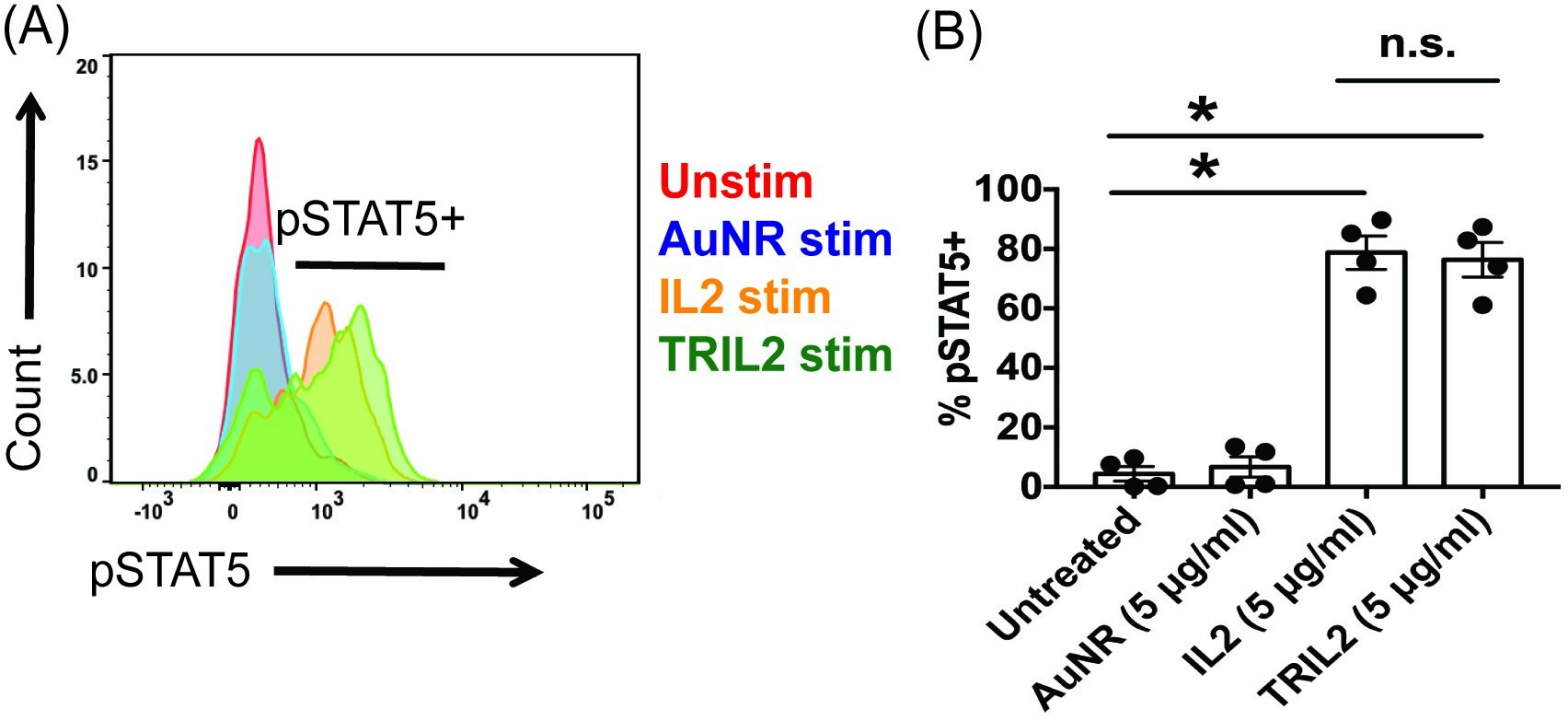
TRIL2 can functionally stimulate pSTAT5 in CD4+ CD25+ Foxp3+ Tregs. **(A)** TRIL2 stimulated CD4+ CD25+ Tregs to upregulate pSTAT5; black horizontal bar indicates pSTAT5+ cells. **(B)** Quantification of pSTAT5. The flow data are gated on the percent live. The data are representative of 2 independent experiments; n = 4 per group. The data is represented as scatter dot plots, where error bars represent the standard error of the mean and *p ≤ 0.05 is significant as determined by a two-sided t test.

Next, we examined if TRIL2 was functionally active by assessing whether it can functionally induce phosphorylated STAT5 (pSTAT5), an important signaling molecule downstream of the IL2R. We stimulated CD25+ Tregs with either IL2 or TRIL2 and found that TRIL2 was able to promote pSTAT5 to the same extent as the IL2 controls (Fig 5A and 5B). However, notably, TRIL2 was able to induce pSTAT5 at a much lower dose than the IL2 controls. Uv-Vis allowed us to calculate that the IL2 loading efficiency was ~5% (S6 Fig). Thus, for every 100 μg of AuNR, 5 μg of IL2 was loaded. Using 5 μg of TRIL2 was equivalent to a 5-fold lower dosage than 5 μg of IL2 alone (Fig 5 and S6 Fig). Thus, even with a 5-fold lower dose of IL2, TRIL2 was able to produce equivalent induction of pSTAT5 (Fig 5) and enhance CD4+ Foxp3+ Tregs via the IL2-pSTAT5 pathway.

In our LDH experiments, we wanted to determine if the differences we were observing were due to AuNR-mediated cytotoxicity of different immune cell subsets. LDH assesses metabolic activity, which allows it to also serve as an indirect measurement of cell viability, as only viable cells are able to metabolize. In order to further examine if AuNRs were cytotoxic to immune cells, we utilized the live dead marker FVD APC-eFluor 780. Incorporation of FVD enables discrimination between live and dead cells. Using FVD and flow cytometry, we confirmed that treatment with up to 100 μg/ml of AuNR and AuNR/Ag did not induce cell death by 24 hours, based on percentages and total cell numbers of splenocytes (S8 Fig). However, we found that at 72 hours, there was a statistical difference (as determined by ANOVA) in the percent of live and total live splenocytes, which may account for the differences and changes in percent CD11c+ MHC class II+ DCs, CD8 T cells, and Tregs (S8 Fig). Notably, at 72 hours, when we compared untreated CD4+ T cells with AuNR- or AuNR/Ag-treated CD4 T cells using a student T test, we found no such differences (S9 Fig). In addition, the total number of CD4+ Foxp3+ Tregs appeared to be no different between treatment groups in the early time points, but differences were seen in later time points (S10 Fig).

## Discussion

In order to develop and use AuNRs as immune targeting vehicles, we must fully delineate how unfunctionalized NRs impact subsets of the immune system. To support this goal, we used an *in vitro* system to study direct exposure of AuNRs to immune cell subsets. We found that in the early time points and up to concentrations of 100 μg/ml, AuNR and AuNR/Ag have minimal impact on splenocyte viability. Based on our previous findings, we felt that 100 μg/ml would be ideal in terms of balance with cytotoxicity and immune impact [46]. We cultured splenocytes and exposed them to 100 μg/ml of AuNR and AuNR/Ag. Subsequently, we assessed the kinetics and dynamics (up to 72 hours) of different immune cell subsets: DCs and T cells. We found that percentages of DCs, as gated by their expression of CD11c and MHC class II, were increased. However, we did not find a difference in pDCs (B220+) and cDCs: CD8+ or CD11b+. This may mean that, overall, AuNR- and AuNR/Ag-treated DC subsets up-regulated CD11c and MHC class II on the surfaces of their cells. However, using an established immune adjuvant was still more effective for increasing frequencies of CD11c+ MHC class II+ DCs [47].

Next, we investigated the impact of AuNR and AuNR/Ag on CD4 and CD8 T cells. We found that compared to AuNR, AuNR/Ag was able to increase CD8+ T cell frequencies within 72 hours (Fig 2). This increase did not correlate with an activation of CD44 and CCR7 expression. Interestingly, AuNR and AuNR/Ag treated cells increased frequencies in CD8+ CD69+ cells (S5 Fig). Though there appeared to be fewer CD8+ IFNγ+ T cells in the AuNR/Ag-treated cells, this did not impact the ability of CD8+ T cells to produce IFNγ, as there was no statistical difference between untreated cells (S5 Fig). Though the AuNR treated cells did impact the production of CD8+ IFNγ+ T cells (S5E Fig). When we examined the impact of AuNR and AuNR/Ag on CD4 T cells, we found no difference in the frequencies of CD4+ T cells at 24 and 72 hours. However, when we examined the Thelper subset CD4+ Foxp3+ Tregs, we found that AuNR significantly increased CD4+ Foxp3+ Tregs by 72 hours (Fig 3C).

Currently, it is unknown how unfunctionalized AuNR and AuNR/Ag influence or interact with distinct immune cell subsets. However, since AuNR and AuNR/Ag appear to differentially impact the production of CD8+ IFNγ+ T cells, it is possible that Thelper-induced mechanisms exist that would explain the differences we see. However, when we stained for IL4, IL6, and IL10, we did not see any differences between the untreated, AuNR-treated, and AuNR/Ag-treated samples in the production of these cytokines (data not shown). However, it may be that the Ag coating is a playing a role in promoting these differences. Though this remains to be further investigated. Thus, the mechanisms of AuNR’s influence on the immune system may be much more complex than initially thought, demanding a more in-depth investigation of individual immune subsets, their development, and mechanisms of homeostasis [48, 49].

Having a better understanding of how AuNRs can impact immune subsets, specifically CD4+ Foxp3+ Tregs, we conjugated IL2 to AuNRs, creating TRIL2. TRIL2 was able to promote CD25+ CD4+ Foxp3+ Tregs and stimulate the IL2-pSTAT5 pathway. Notably, we found that TRIL2 was equally as potent as a higher dose of IL2 alone as TRIL2 as able to induce pSTAT5 at much lower doses. This is attributed to the loading efficiency of IL2, which was 5 μg of IL2 per 100 μg of AuNRs. Thus, using AuNRs as carriers for cytokines such as IL2 may make it possible to load IL2 concentrations at a much lower-fold dose. This would have important consequences in immunotherapy, as high-dose IL2 therapy can result in toxicity [50]. Next, this promising implication needs to be fully investigated in murine models of autoimmune diseases.

Interestingly, we did not utilize any cytokines or T cell agonists (α-CD3, α-CD28) to enhance immune cell survival. Perhaps the differences we see are due to some immune subsets responding better to AuNRs than others. However, the cause is more likely to be AuNR-mediated cell death, which could alter the percentages of other immune subsets. At first glance, the AuNR and AuNR/Ag appeared to be nontoxic, as the LDH assay and FVD exclusion did not indicate any changes in cell viability in the early time points (S8–S10 Figs). However, by 72 hours, the FVD dye exclusion technique indicated differences in cytotoxicity, which could account for the percent changes in our immune subsets. Notably in our cell proliferation assays, we found that both AuNR and AuNR/Ag did not appear to induce proliferation compared to the unstimulated splenocytes. However the proliferation of splenocytes stimulated with IL2 and treated with AuNRs and AuNR/Ag appeared to be synergistic (S11 Fig, top row). Thus, the changes in percentages of immune cells in response to AuNR or AuNR/Ag treatment may be a consequence of AuNR-induced survival or death.

It may be that the immune cells treated with AuNRs are producing IL2, which could give T cells such as CD8 and CD4+ Foxp3+ Tregs a survival advantage [29]. Interleukin-2, a potent T cell growth factor, is one of the cytokines involved in CD4+ Foxp3+ Treg development [12, 13, 51]. We have shown that the main source of IL2 is T cell derived [44]. However, DCs can also produce IL2. Importantly, *Dykman and Khlebtsov 2017*, have reported that immune cells treated with AuNPs can produce IL2 [29]. Hence, though it is possible that AuNR-mediated IL2 derived from a T cell and DC source may be involved in mediating IL-2-dependent expansion of regulatory T lymphocytes, we think this scenario is unlikely due to the decrease in cell viability of the immune cells (S8–S10 Figs). Moreover, proliferation of splenocytes treated without IL2 was comparable between AuNR and AuNR/Ag, indicating that proliferation may not play a major role in their expansion (S11 Fig). However, this does not account for the differences we see in the percent CD8+ CD69+ cells and remains to be investigated.

It is thought that AuNPs interact with cell surface receptors, which in turns induce the production of cytokines [52]. This implies that there is some specificity in receptor-mediated internalization of gold nanoparticles. However, since the AuNRs utilized in our studies (except for TRIL2) were not conjugated with ligands, this type of receptor-mediated internalization is unlikely. Our findings, both herein and previously published, suggest that this interaction is non-specific and that any uptake of the AuNRs is independent of receptor-mediated endocytosis [33]. Furthermore, at the early time point, we did not see a change in cell granularity, as analyzed by side scatter area (S12 Fig). It is noteworthy that changes in cellular granularity were reported after macrophages internalized AuNRs [53].

We also found that AuNRs had a varied impact on immune function based on the production of IFNγ, STAT5, and Foxp3. At the early time point, AuNRs did not inhibit production of IFNγ. However, after 72 hours, there was a statistical difference in the production of IFNγ by AuNR-treated CD8+ T cells (S5 Fig). Notably, AuNRs did not impair the induction and function of the transcription factor STAT5, nor did they impair Foxp3 production (Figs 4 and 5). In Treg development, Foxp3 induction depends on the 2-step model of Treg differentiation (one from the TCR and the second from cytokine signaling), which may indicate that TCR signaling and function are not impacted by treatment with AuNRs [14, 43]. This means that AuNRs could impact IFNγ production but not STAT5 and Foxp3 induction.

Our findings suggest that unfunctionalized AuNRs have a nonspecific impact on cultured splenocytes, increasing the frequency of myeloid DCs, CD8+ cells, and Tregs in a resting *in vitro* assay. The significance of this data is three-fold: 1. it offers a more complete picture of how continuous exposure to AuNRs impacts different immune cell subsets of the immune system; 2. it suggests that AuNRs could enhance Tregulatory cells and play a role in immunosuppression; and 3. it provides strong evidence that the choice of AuNRs matters when trying to enhance or target a particular immune cell of interest, as different nanomaterials impact different immune cells in different ways.

Future work will investigate how NP-mediated activation may alter other immune cell function or synergize with stimulatory conditions. Moreover, it is possible that AuNPs persist *in vivo* and in tissues and organs systems indefinitely, making it crucial to further investigate the continuous exposure of AuNRs to immune cells, particularly *in vivo*. In addition, future work should investigate the duration of AuNP-induced immune effects and their long-term interaction with immune cells, if any.

## Conclusion

In summary, treatment with AuNR and AuNR/Ag did not result in different impacts within the DC subsets but, overall, enhanced CD11c+ MHC class II+ DCs. We did see minor differences within the T cell compartment (CD4 versus CD8 T cells) and on their activation state and function. In addition, we found that AuNRs enhanced the Thelper subset CD4+ Foxp3+ Tregs, while AuNR/Ag did not. Our results show that choosing a nanoparticle is not as simple it appears and requires careful analysis, and that the way NPs interact with immune cells and their subsets is more complex than initially thought.

The results of our studies indicate that no two NPs are alike in their ability to impact immune subsets. In order to use AuNPs in immune targeting, we must fully establish a basic understanding of how they can influence the immune system. The choice of NP will depend on which cells of the immune system it can enhance, not just NP characteristics, size, surface charge, and functionalization. In order to be used safely and effectively in nanomedicine, a NP must be analyzed for its impact on individual immune subsets.

## Supporting information

S1 Fig AuNRs are nontoxic up to ~100 μg/ml. Cytotoxicity was assessed by LDH assay after treating primary immune cells derived from splenocytes with up to ~250 μg/ml of AuNR (left panel) and AuNR/Ag (right panel) for 24 hours. The data is representative of 3 independent experiments with at least 3 technical replicates, error bars represent the standard error of the mean.
(TIF)

S2 Fig AuNR and AuNR/Ag impact different DC subsets. CD11c+ MHC classII+ B220+ DCs **(A)** and **(B),** CD11c+ MHC classII+ CD11b+ DCs **(C)** and **(D)**, and CD11c+ MHC classII+ CD8+ DCs **(E)** and **(F).** Left column is indicative of 24 hours and right column is 72 hours. The data shown are based on the live gate. The data is shown as scatter dot plots, where the data is representative of 3 independent experiments, error bars represent the SEM, and *p ≤ 0.05 is significant as determined by a two-sided t test.
(TIF)

S3 Fig AuNRs impact CD4 and CD8 T cells differentially. AuNR and AuNR/Ag impact CD4 and CD8 T cells at 24 hours, **(A)** CD4+ T cells and, **(B)** CD8+ T cells. The data is based on the live gate and is shown as scatter dot plots, where the data is representative of 3 independent experiments, error bars represent the SEM, and *p ≤ 0.05 is significant as determined by a two-sided t test.
(TIF)

S4 Fig AuNR and AuNR/Ag impact T cell activation differently. Splenocytes were either untreated or treated with LPS (5 μg/ml) or 100 μg/ml of AuNR and AuNR/Ag for 24 hours (left column) or 72 hours (right column), and flow cytometry was performed. Combined data represent 3 independent experiments with at least 3 replicates per experiment; the data is gated on the live gate. The data is shown as scatter dot plots, where the error bars represent the standard error of the mean (SEM) and *p ≤ 0.05 is significant as determined by a two-sided t test.
(TIF)

S5 Fig Differential impact of AuNRs on CD8 T cell activation. CD8 T cell activation as investigated by surface staining of **(A)** CD69 at 24 hours, **(B)** CD69 at 72 hours, **(C)** CD8+ IFNγ+ at 72 hours, **(D)** CD8+ IFNγ+ at 24 hours, and **(E)** CD8+ IFNγ+ at 72 hours. The data is shown as a scatter dot plot, where data is representative of 3 independent experiments based on the live gate; error bars represent the standard error of the mean (SEM) and *p ≤ 0.05 is significant as determined by a two-sided t test.
(TIF)

S6 Fig IL2 loading efficiency as examined by Uv-Vis. **(A)** Uv-Vis-NIR spectra of TRIL2, IL2, and AuNR/PEG. **(B)** Uv-Vis-NIR spectra of different concentrations of TRIL2 were used to generate the wavelength vs. absorbance graph at ~279 nm, which was utilized to generate **(C)** a concentration vs. absorbance graph.
(TIF)

S7 Fig SEM and AFM of AuNRs and TRIL2. SEM image of **(A)** AuNR without PEG coating and **(B)** TRIL2. AFM islet: AFM height image of AuNRs (top right) and TRIL2 (bottom left), captured by scanning a 1-micron area. The AFM images were acquired and analyzed with NanoScope Analysis software.
(TIF)

S8 Fig Percent of live and total number of splenocytes after AuNR and AuNR/Ag treatment. Whole splenocytes were untreated, treated with LPS (5 μg/ml, positive control), treated with 100 μg/ml AuNR, or treated with 100 μg/ml AuNR/Ag for up to 72 hours, **(A)(B)** 24 hours and **(C)(D)** 72 hours. At 24 or 72 hours, cells were harvested and stained for DC markers **(E)** and **(F).** The flow data are representative of 3 independent experiments based on the live gate with at least 3 technical replicates in each. The data is shown as scatter dot plots, where error bars represent the standard error of the mean (SEM), ANOVA with a Bartlett’s test, and *p ≤ 0.05 is significant.
(TIF)

S9 Fig Total number of CD4 and CD8+ T cells after treating with AuNRs. Whole splenocytes were untreated, treated with LPS (5 μg/ml), treated with 100 μg/ml AuNR, or treated with 100 μg/ml AuNR/Ag for up to 72 hours. Total number of CD4+ T cells (**A)** 24 hours and **(B)** 72 hours. Total number of CD8+ T cells **(C)** 24 hours and **(D)** 72 hours. The flow data are representative of 3 independent experiments with at least 3 technical replicates in each; the data is based on the live gate. The data is shown as scatter dot plots, where error bars represent the standard error of the mean (SEM), ANOVA with a Bartlett’s test, and *p ≤ 0.05 is significant.
(TIF)

S10 Fig The percent of live and total number of CD4+ Foxp3+ Tregs after treating with AuNRs. Whole splenocytes were untreated, treated with LPS (5 μg/ml), treated with 100 μg/ml AuNR, or treated with 100 μg/ml AuNR/Ag for up to 72 hours. The percent of live and total number of CD4+ Foxp3+ Tregs after **(A)** 24 hours and **(B)** 72 hours. The flow data are representative of 3 independent experiments with at least 3 technical replicates in each based on the live gate. The data is shown as scatter dot plots where error bars represent the standard error of the mean (SEM), ANOVA with a Bartlett’s test, and *p ≤ 0.05 is significant.
(TIF)

S11 Fig AuNR and AuNR/Ag did not induce proliferation of splenocytes after 72 hours. Splenocytes were stained with CPD eFlour 670 and then stimulated with **(A)** IL2 (5 μg/ml) or **(B)** without IL2. In panel **(A)** and **(B),** splenocytes were treated with Conacanvalin A (5 μg/ml), AuNR (100 μg/ml), and AuNR/Ag (100 μg/ml) for up to 72 hours. After 72 hours, the cells were harvested, washed, and prepared for flow cytometry. The data is based on the live gate. The green histogram represents cell proliferation based on eFlour 670 dilution over time. The data is representative of 2 independent experiments, n = 6 total.
(TIF)

S12 Fig AuNRs do not impact the granulosity of the whole splenocytes. Whole splenocytes were untreated, treated with LPS (5 μg/ml), treated with 100 μg/ml AuNR, or treated with 100 μg/ml AuNR/Ag for up to 72 hours. The scatter dot plot is representative of 3 independent experiments with at least 3 technical replicates.
(TIF)
