# Supplementary figures and images for: Gold nanorods enhance different immune cells and allow for efficient targeting of CD4+ Foxp3+ Tregulatory cells
Source: PLoS One. 2021 Aug 30;16(8):e0241882. doi: 10.1371/journal.pone.0241882 (PMC8404976; doi:10.1371/journal.pone.0241882)

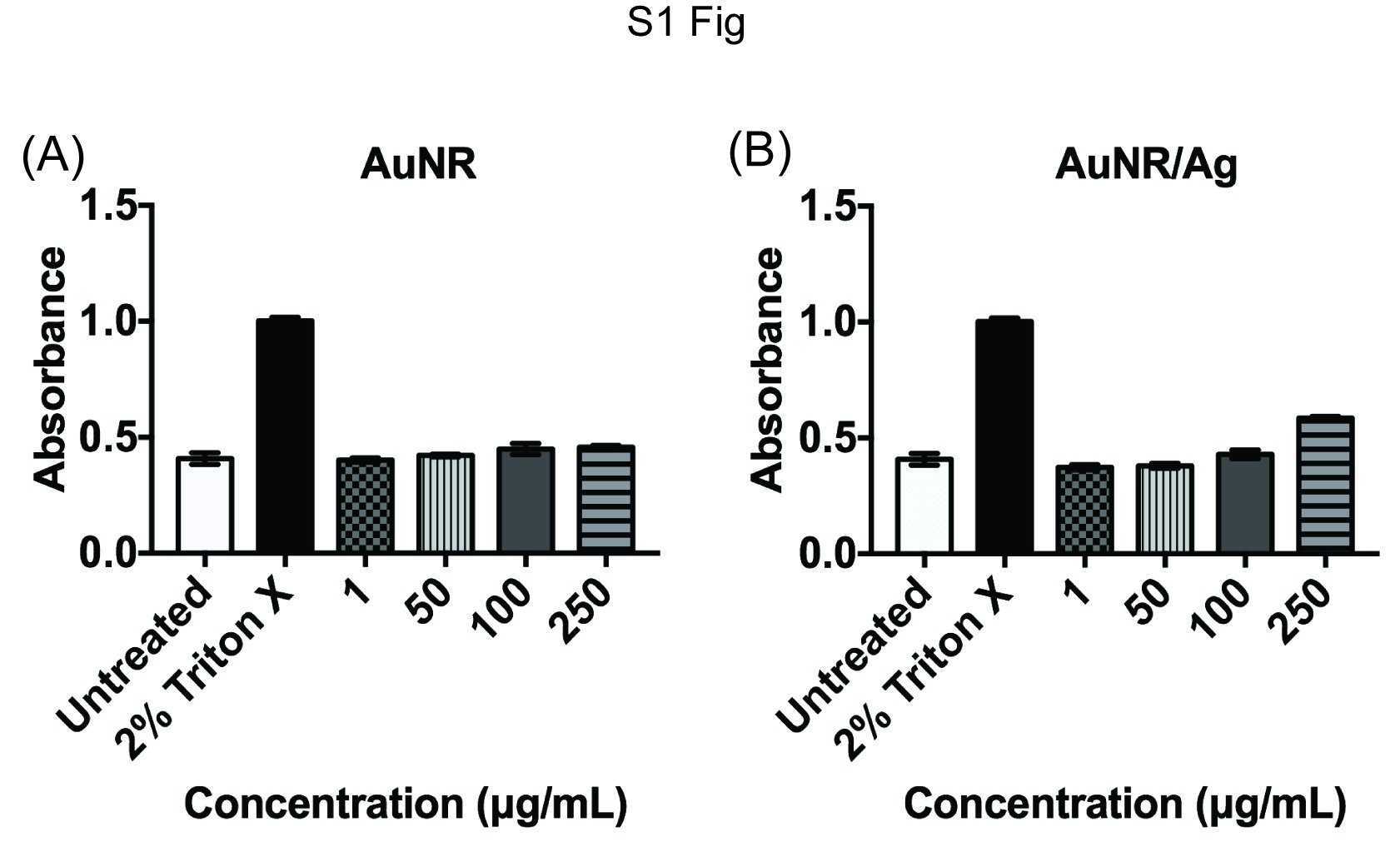

Supplement: S1 Fig — Cytotoxicity was assessed by LDH assay after treating primary immune cells derived from splenocytes with up to ~250 μg/ml of AuNR (left panel) and AuNR/Ag (right panel) for 24 hours. The data is representative of 3 independent experiments with at least 3 technical replicates, error bars represent the standard error of the mean. (TIF) [file pone.0241882.s001.tif]

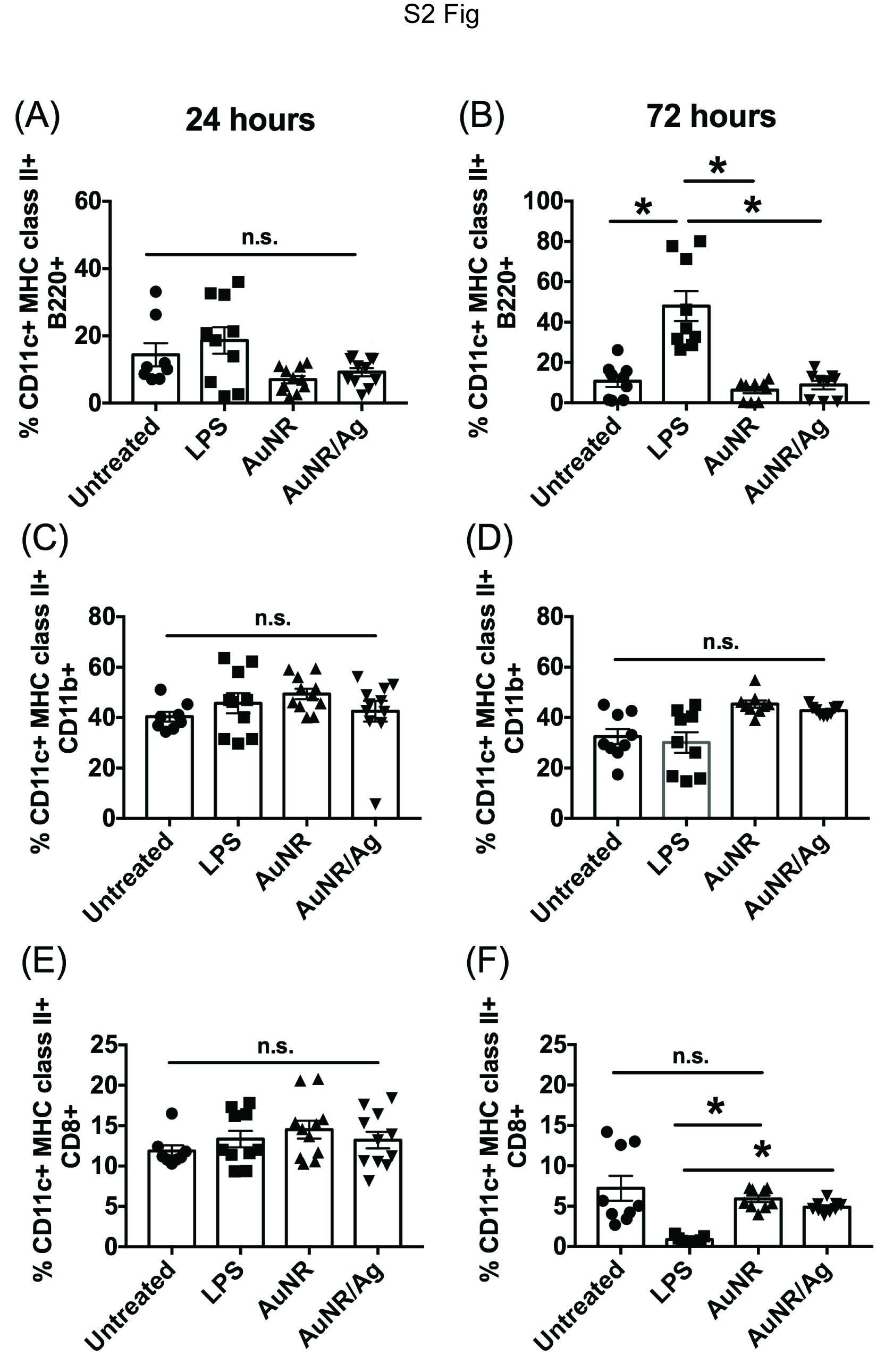

Supplement: S2 Fig — CD11c+ MHC classII+ B220+ DCs (A) and (B), CD11c+ MHC classII+ CD11b+ DCs (C) and (D), and CD11c+ MHC classII+ CD8+ DCs (E) and (F). Left column is indicative of 24 hours and right column is 72 hours. The data shown are based on the live gate. The data is shown as scatter dot plots, where the data is representative of 3 independent experiments, error bars represent the SEM, and *p ≤ 0.05 is significant as determined by a two-sided t test. (TIF) [file pone.0241882.s002.tif]

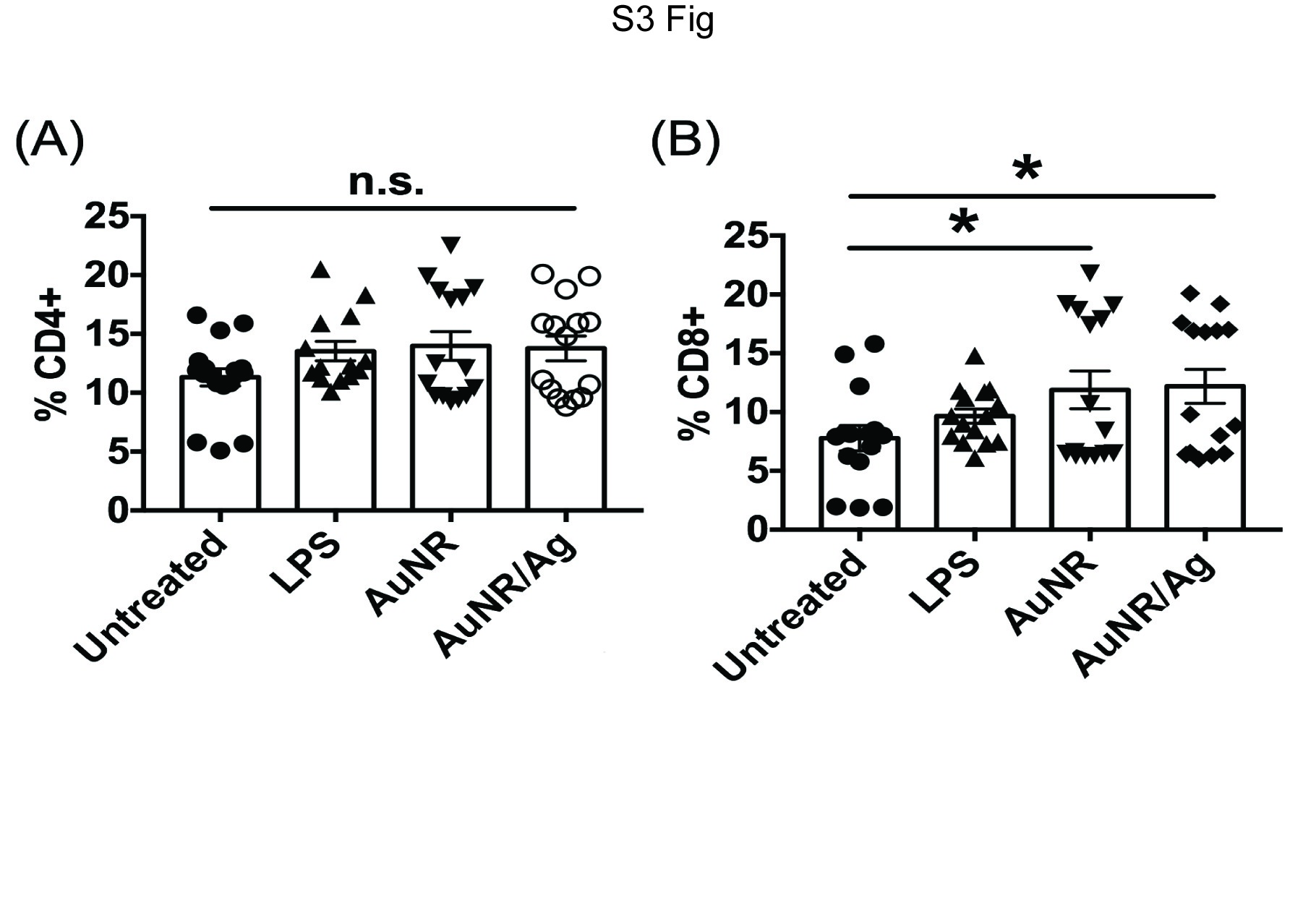

Supplement: S3 Fig — AuNR and AuNR/Ag impact CD4 and CD8 T cells at 24 hours, (A) CD4+ T cells and, (B) CD8+ T cells. The data is based on the live gate and is shown as scatter dot plots, where the data is representative of 3 independent experiments, error bars represent the SEM, and *p ≤ 0.05 is significant as determined by a two-sided t test. (TIF) [file pone.0241882.s003.tif]

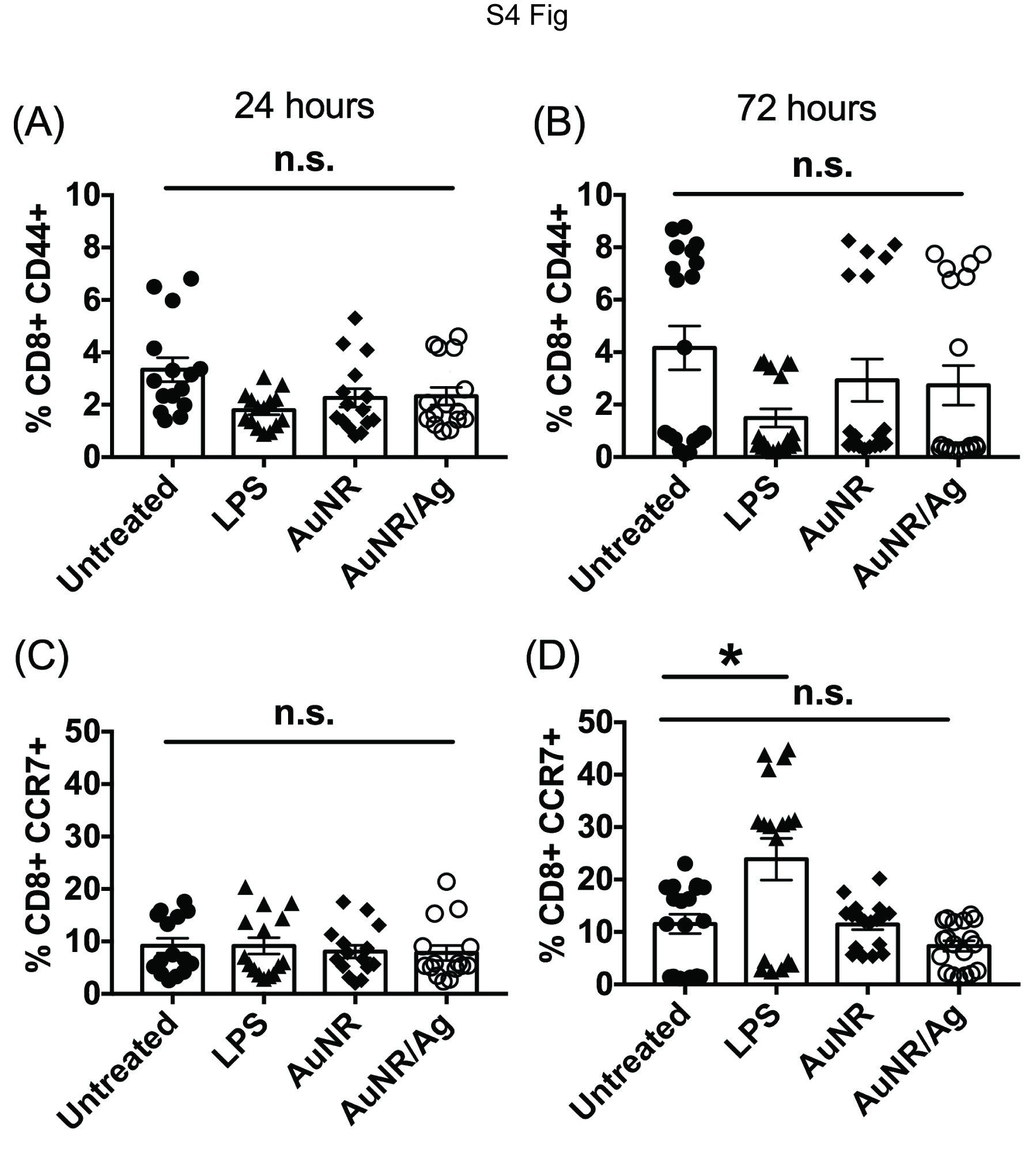

Supplement: S4 Fig — Splenocytes were either untreated or treated with LPS (5 μg/ml) or 100 μg/ml of AuNR and AuNR/Ag for 24 hours (left column) or 72 hours (right column), and flow cytometry was performed. Combined data represent 3 independent experiments with at least 3 replicates per experiment; the data is gated on the live gate. The data is shown as scatter dot plots, where the error bars represent the standard error of the mean (SEM) and *p ≤ 0.05 is significant as determined by a two-sided t test. (TIF) [file pone.0241882.s004.tif]

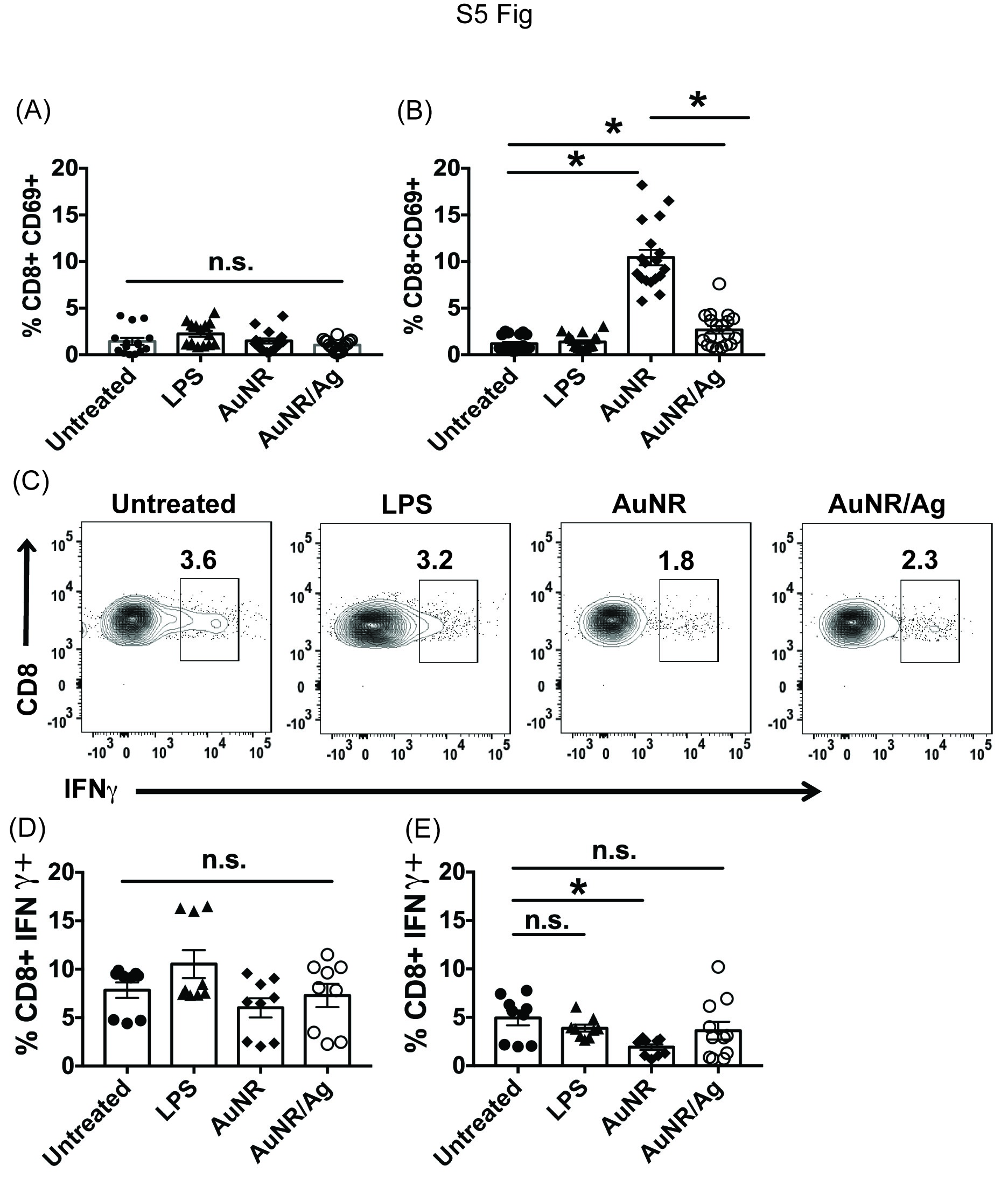

Supplement: S5 Fig — CD8 T cell activation as investigated by surface staining of (A) CD69 at 24 hours, (B) CD69 at 72 hours, (C) CD8+ IFNγ+ at 72 hours, (D) CD8+ IFNγ+ at 24 hours, and (E) CD8+ IFNγ+ at 72 hours. The data is shown as a scatter dot plot, where data is representative of 3 independent experiments based on the live gate; error bars represent the standard error of the mean (SEM) and *p ≤ 0.05 is significant as determined by a two-sided t test. (TIF) [file pone.0241882.s005.tif]

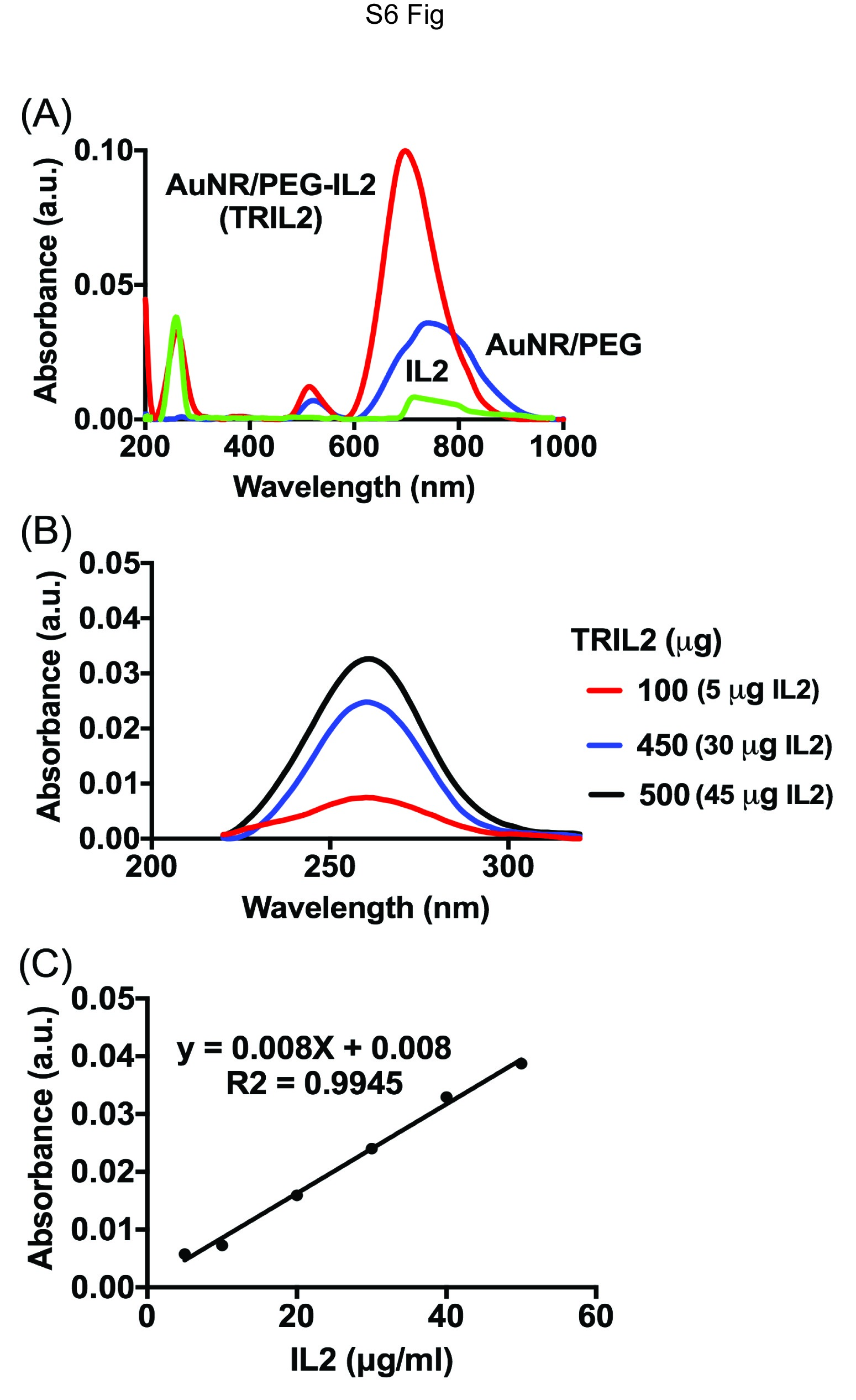

Supplement: S6 Fig — (A) Uv-Vis-NIR spectra of TRIL2, IL2, and AuNR/PEG. (B) Uv-Vis-NIR spectra of different concentrations of TRIL2 were used to generate the wavelength vs. absorbance graph at ~279 nm, which was utilized to generate (C) a concentration vs. absorbance graph. (TIF) [file pone.0241882.s006.tif]

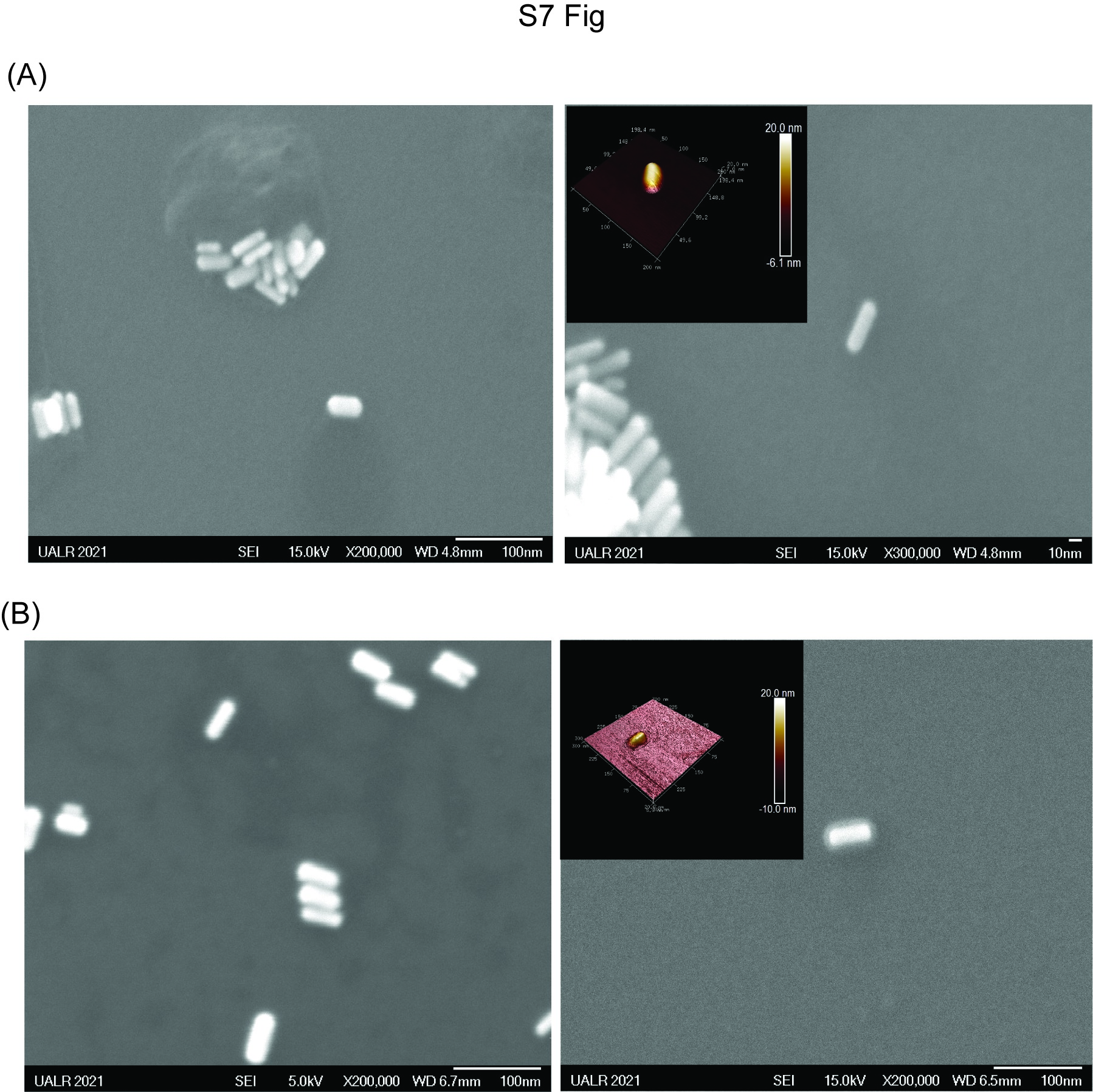

Supplement: S7 Fig — SEM image of (A) AuNR without PEG coating and (B) TRIL2. AFM islet: AFM height image of AuNRs (top right) and TRIL2 (bottom left), captured by scanning a 1-micron area. The AFM images were acquired and analyzed with NanoScope Analysis software. (TIF) [file pone.0241882.s007.tif]

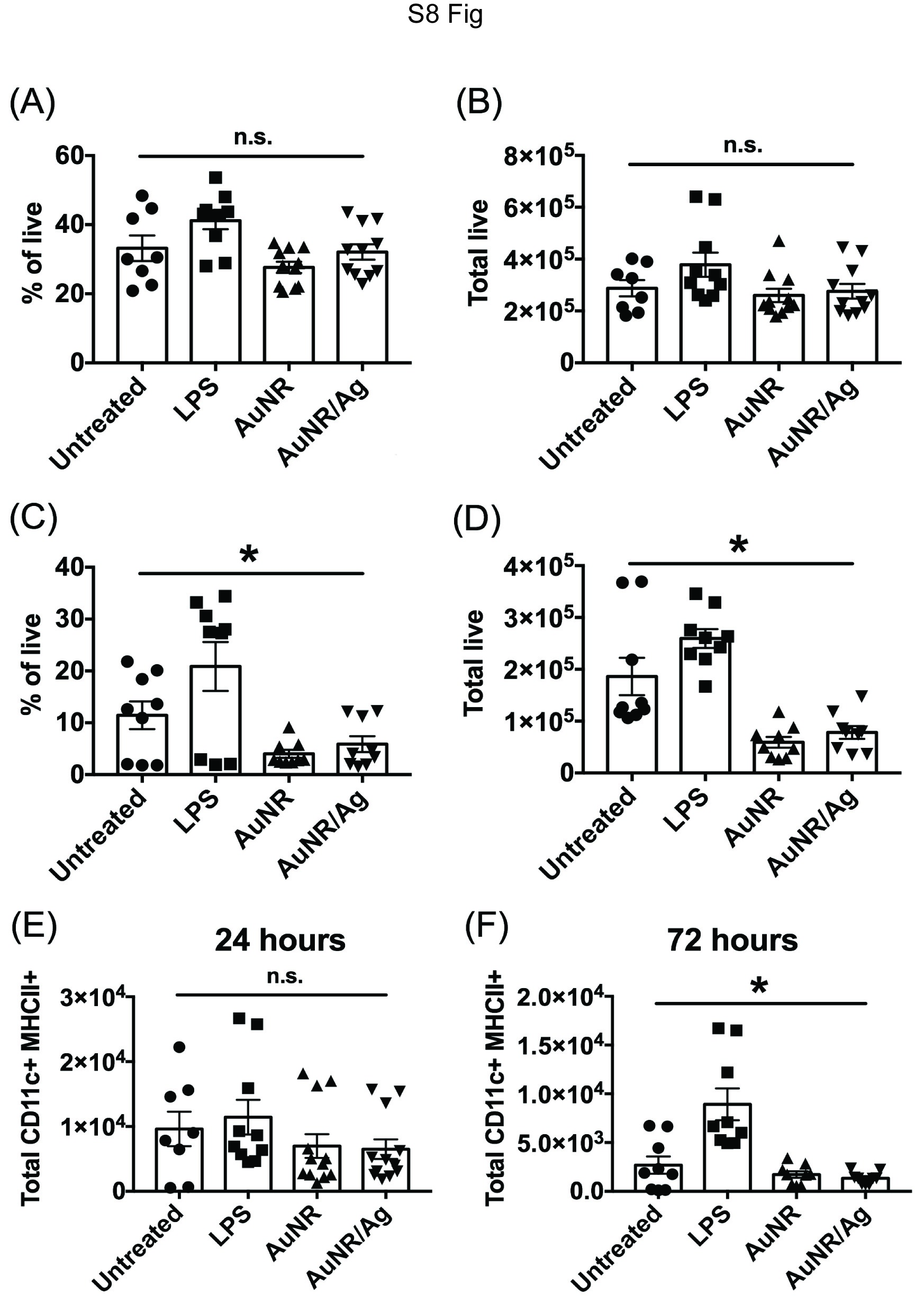

Supplement: S8 Fig — Whole splenocytes were untreated, treated with LPS (5 μg/ml, positive control), treated with 100 μg/ml AuNR, or treated with 100 μg/ml AuNR/Ag for up to 72 hours, (A)(B) 24 hours and (C)(D) 72 hours. At 24 or 72 hours, cells were harvested and stained for DC markers (E) and (F). The flow data are representative of 3 independent experiments based on the live gate with at least 3 technical replicates in each. The data is shown as scatter dot plots, where error bars represent the standard error of the mean (SEM), ANOVA with a Bartlett’s test, and *p ≤ 0.05 is significant. (TIF) [file pone.0241882.s008.tif]

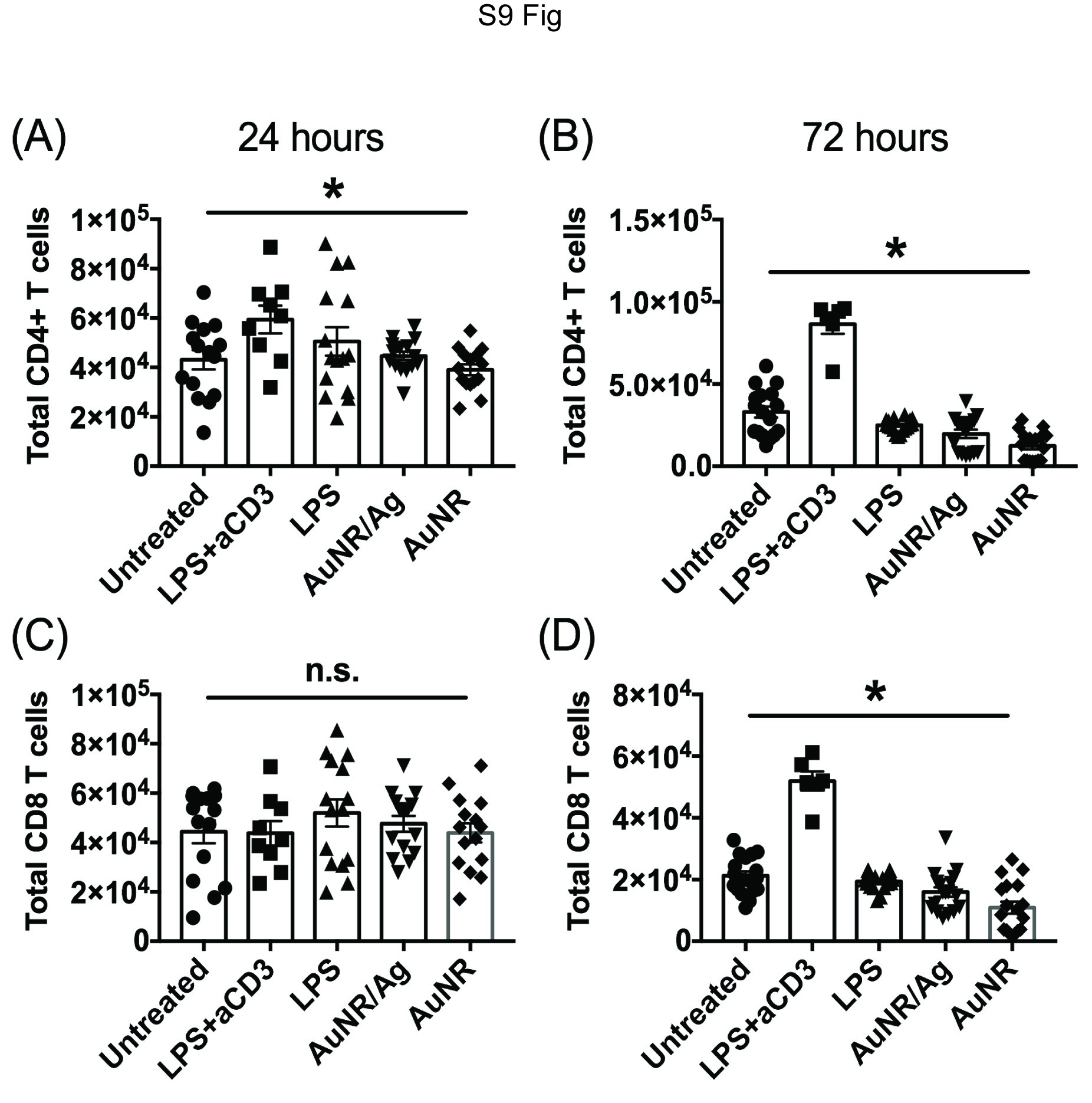

Supplement: S9 Fig — Whole splenocytes were untreated, treated with LPS (5 μg/ml), treated with 100 μg/ml AuNR, or treated with 100 μg/ml AuNR/Ag for up to 72 hours. Total number of CD4+ T cells (A) 24 hours and (B) 72 hours. Total number of CD8+ T cells (C) 24 hours and (D) 72 hours. The flow data are representative of 3 independent experiments with at least 3 technical replicates in each; the data is based on the live gate. The data is shown as scatter dot plots, where error bars represent the standard error of the mean (SEM), ANOVA with a Bartlett’s test, and *p ≤ 0.05 is significant. (TIF) [file pone.0241882.s009.tif]

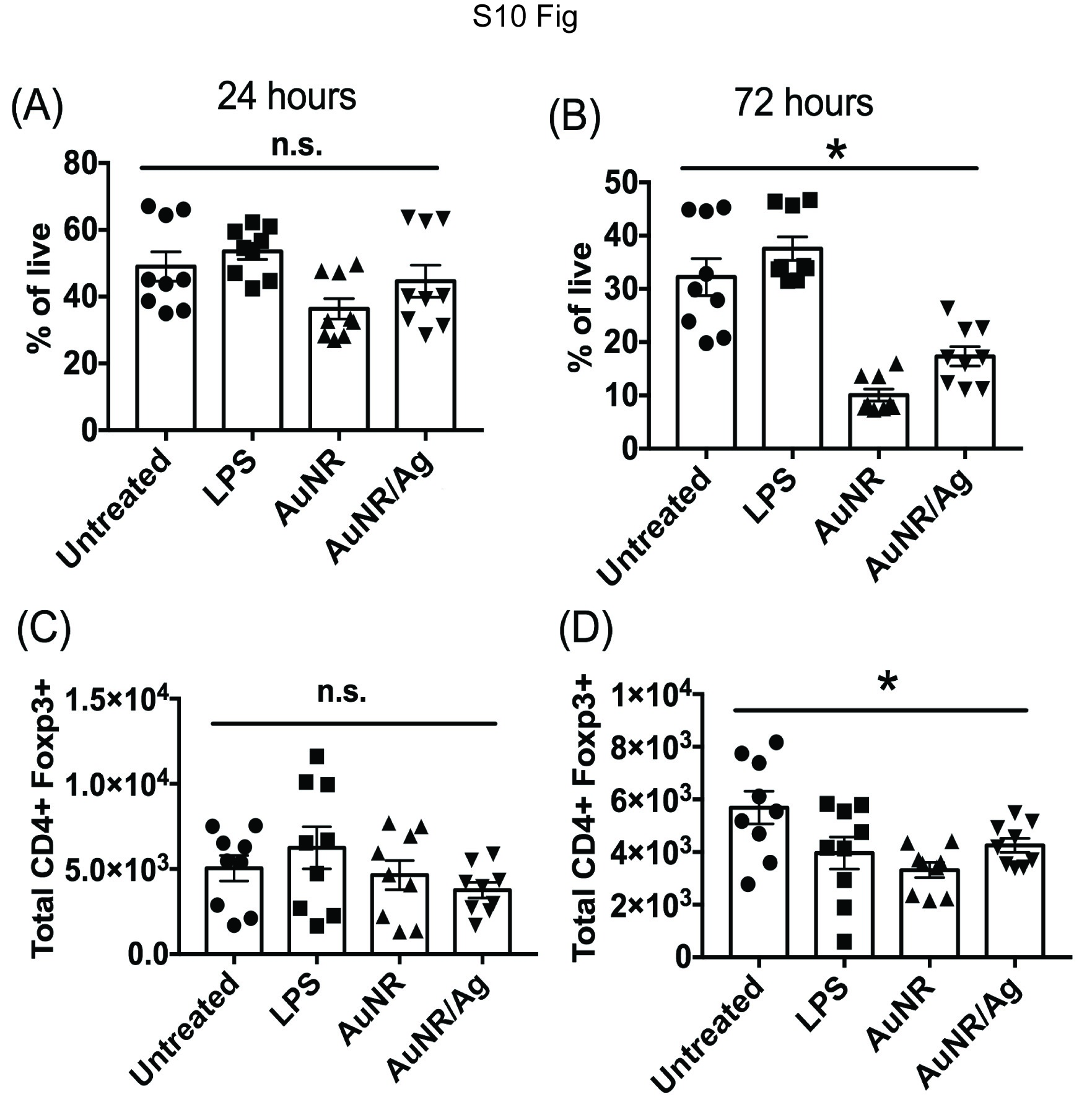

Supplement: S10 Fig — Whole splenocytes were untreated, treated with LPS (5 μg/ml), treated with 100 μg/ml AuNR, or treated with 100 μg/ml AuNR/Ag for up to 72 hours. The percent of live and total number of CD4+ Foxp3+ Tregs after (A) 24 hours and (B) 72 hours. The flow data are representative of 3 independent experiments with at least 3 technical replicates in each based on the live gate. The data is shown as scatter dot plots where error bars represent the standard error of the mean (SEM), ANOVA with a Bartlett’s test, and *p ≤ 0.05 is significant. (TIF) [file pone.0241882.s010.tif]

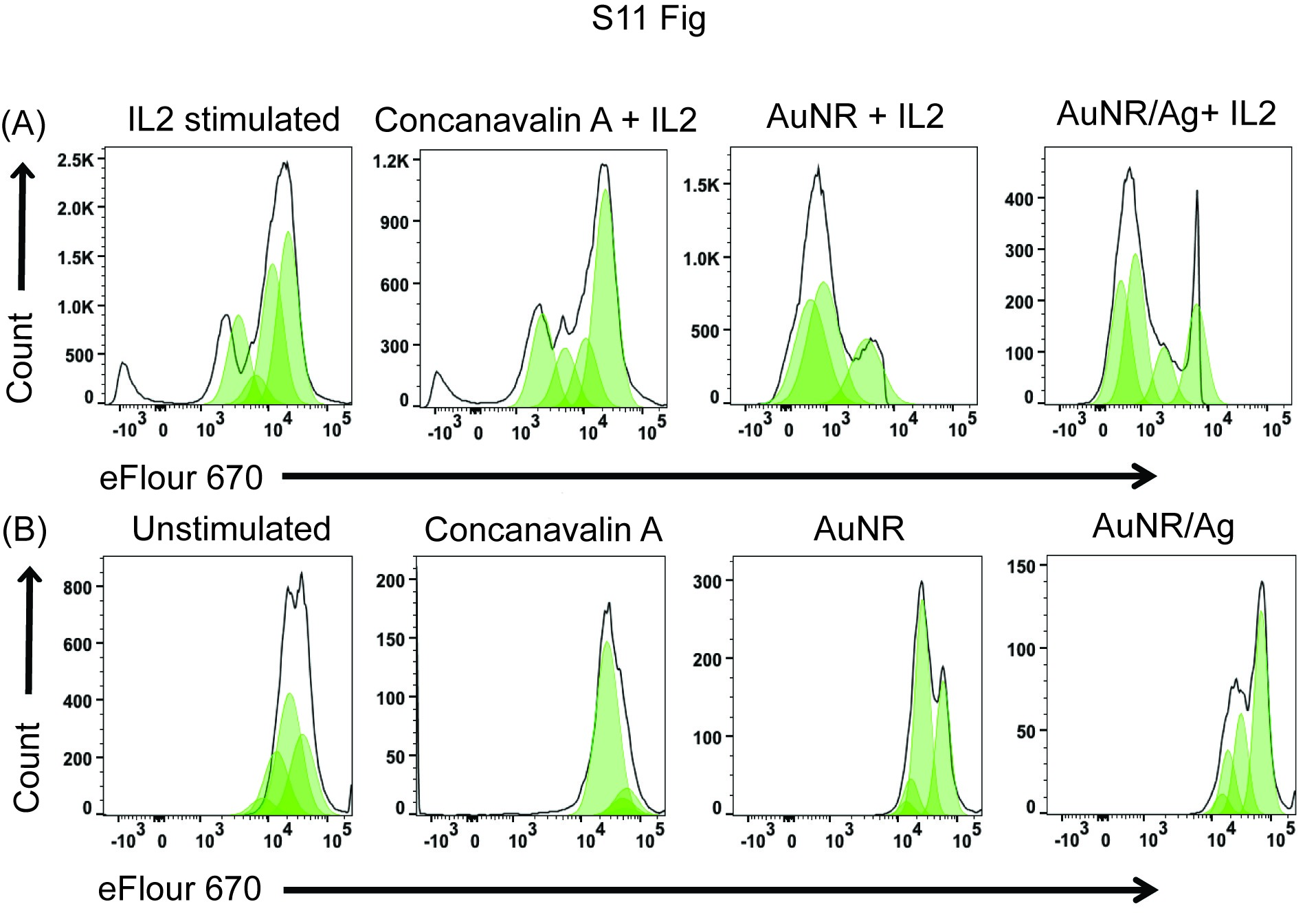

Supplement: S11 Fig — Splenocytes were stained with CPD eFlour 670 and then stimulated with (A) IL2 (5 μg/ml) or (B) without IL2. In panel (A) and (B), splenocytes were treated with Conacanvalin A (5 μg/ml), AuNR (100 μg/ml), and AuNR/Ag (100 μg/ml) for up to 72 hours. After 72 hours, the cells were harvested, washed, and prepared for flow cytometry. The data is based on the live gate. The green histogram represents cell proliferation based on eFlour 670 dilution over time. The data is representative of 2 independent experiments, n = 6 total. (TIF) [file pone.0241882.s011.tif]

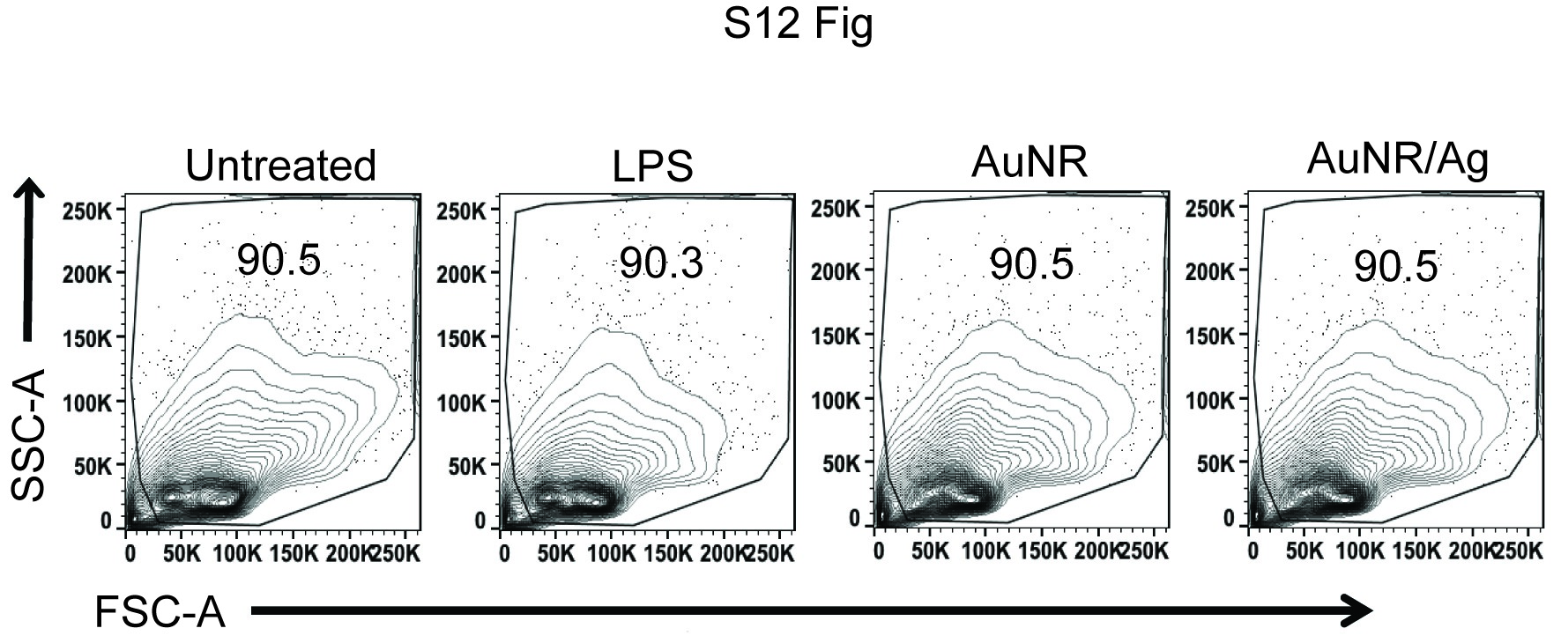

Supplement: S12 Fig — Whole splenocytes were untreated, treated with LPS (5 μg/ml), treated with 100 μg/ml AuNR, or treated with 100 μg/ml AuNR/Ag for up to 72 hours. The scatter dot plot is representative of 3 independent experiments with at least 3 technical replicates. (TIF) [file pone.0241882.s012.tif]
